# Competing Kinetic Consequences of CO_2_ on the Oxidative Degradation of Branched Poly(ethylenimine)

**DOI:** 10.1021/jacs.4c08126

**Published:** 2024-08-30

**Authors:** Sichi Li, Yoseph Guta, Marcos F. Calegari Andrade, Elwin Hunter-Sellars, Amitesh Maiti, Anthony J. Varni, Paco Tang, Carsten Sievers, Simon H. Pang, Christopher W. Jones

**Affiliations:** † Materials Science Division, Lawrence Livermore National Laboratory, Livermore, California 94550, United States; ‡ School of Chemical & Biomolecular Engineering, Georgia Institute of Technology, Atlanta, Georgia 30332, United States

## Abstract

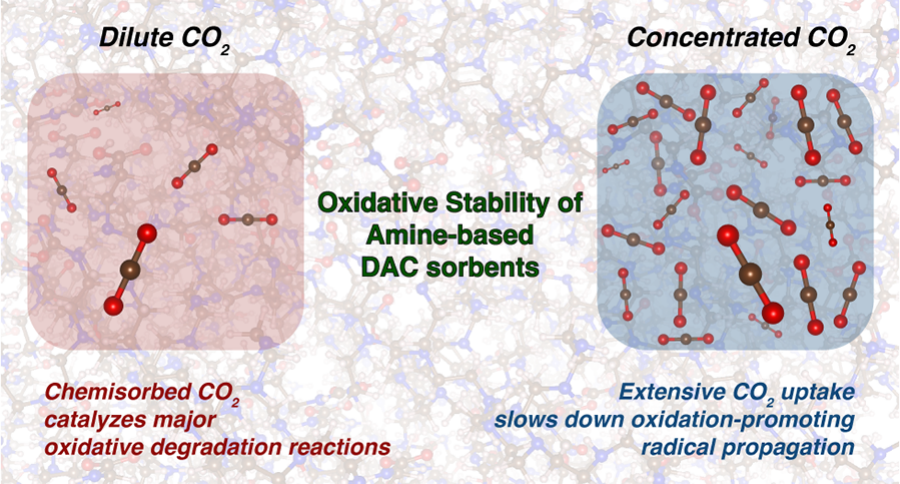

Amine-functionalized porous solid materials are effective sorbents for direct air capture (DAC) of CO_2_. However, they are prone to oxidative degradation in service, increasing the materials cost for widespread implementation. While the identification of oxidation products has given insights into degradation pathways, the roles of some species, like CO_2_ itself, remain unresolved, with conflicting information in the literature. Here, we investigate the impact of CO_2_ on the oxidative degradation of poly(ethylenimine)–alumina (PEI/Al_2_O_3_) sorbents under conditions encompassing a wide range of CO_2_-air mixture compositions and temperatures relevant to DAC conditions, thereby reconciling the conflicting data in the literature. Degradation profiles characterized by thermogravimetric analysis, *in situ* ATR-FTIR, and CO_2_ capacity measurements reveal nonmonotonic effects of CO_2_ concentrations and temperatures on oxidation kinetics. Specifically, 0.04% CO_2_ accelerates PEI/Al_2_O_3_ oxidation more at low temperatures (<90 °C) compared to 1% and 5% CO_2_, but this trend reverses at high temperatures (>90 °C). First-principles metadynamics, machine learning accelerated molecular dynamics simulations, and ^1^H relaxometry experiments show that chemisorbed CO_2_ acid-catalyzes critical oxidation reactions, while extensive CO_2_ uptake reduces PEI branch mobility, slowing radical propagation. These contrasting kinetic effects of CO_2_ explain the complex degradation profiles observed in this work and in prior literature. Collectively, this work highlights the importance of considering atmospheric components in the design of DAC sorbents and processes. Additionally, it identifies the unconstrained branch mobility and local acid environment as two of the major culprits in the oxidation of amine-based sorbents, suggesting potential strategies to mitigate sorbent degradation.

## Introduction

Continuously increasing anthropogenic CO_2_ emissions are the leading contributor to global warming.^1,2^ Accelerated deployment of CO_2_ removal technologies that capture ultradilute CO_2_ at large scales, such as direct air capture (DAC) processes, is critical in mitigating the impact and preventing further environmental damage.^3−5^ Processes based on amine-based solid sorbents are a class of DAC technologies that are achieving industrial-scale implementation owing to their high CO_2_ adsorption capacities, fast CO_2_ uptake, high CO_2_ selectivities, and potential for low cost.^1,6−8^ While these properties are essential for commercial-scale DAC operations, sorbent stability over multiple cycles is one of the most critical factors in determining sorbent lifetime and process costs.^1,9,10^

In DAC, environmental and process parameters play a key role in long-term sorbent stability. Some of the main environmental and process parameters affecting stability are the consistently high ambient O_2_ concentration, the elevated temperature required for regeneration, and the local atmospheric H_2_O concentration. During a typical DAC process using solid amine sorbents, the combination of high O_2_ concentration and high temperatures around the sorbents is avoided. However, when process upsets occur, increased O_2_ concentration during the sorbent regeneration step where the sorbent temperature is the highest can lead to severe oxidative degradation. For example, a recent study reported that exposure to ppm levels of O_2_ during sorbent (benzylamine-based) regeneration can cause higher oxidative degradation than exposure to high O_2_ concentration at intermediate temperatures (56–70 °C).^11^

To date, most studies on amine-functionalized sorbent stability focused on the impact of O_2_ (21–100%) and/or temperature primarily under dry conditions.^12−20^ Such conditions have been used to predict the long-term deactivation at lower temperatures and accelerate data collection, which are crucial since DAC using amine-functionalized sorbents is being deployed. These studies paved the way for the development of a fundamental understanding of the oxidative degradation mechanism^21^ and provided insights for enhancing sorbent stability. Based on these studies, several mitigation techniques have been proposed to prolong sorbent lifetime such as incorporating additives,^22^ functionalization of the aminopolymers,^17,18^ and designing more oxidatively resistant aminopolymers.^23,24^

Although these studies have advanced our understanding of aminopolymer sorbent stability, recent reports have shown that other environmental components, such as CO_2_, also impact the kinetics of sorbent degradation.^25^ Most studies on the role of CO_2_ in aminopolymer sorbent stability have focused on high CO_2_ concentrations, such as flue gas mixtures,^10,20,26^ or have mostly observed the impact of CO_2_ alone on sorbent degradation.^27−31^ For instance, Heydari-Gorji and Sayari reported that CO_2_ protects amine sites on a PEI/SBA-15 sorbent from oxidation, minimizing sorbent degradation and prolonging sorbent lifetime in CO_2_ and O_2_-containing mixtures.^20^ However, these results do not directly translate to DAC conditions. In contrast, focusing on dilute CO_2_ conditions relevant to DAC, Guta et al. reported that the copresence of CO_2_ (0.04%) and O_2_ (21%) accelerates sorbent deactivation, which is contradictory to the sorbent behavior under concentrated CO_2_ conditions.^25^ The mechanistic origin of such a discrepancy in the effect of CO_2_ on PEI-based solid sorbents remains elusive, and reconciling the observed trends is crucial to a comprehensive knowledge of amine sorbent stability, thus enabling improved DAC technology.

To this end, we combined experiments and first-principles modeling to determine the impact of CO_2_ concentration and temperature on the stability of a model aminopolymer sorbent, branched PEI (bPEI) supported on Al_2_O_3_. Using thermogravimetric analysis (TGA) and *in situ* ATR-IR spectroscopy, we investigate the stability of PEI/γ-Al_2_O_3_ by exposing the sorbent to 0.04%, 1%, and 5% CO_2_-air mixtures under dry conditions for 18 h at varying temperatures (30–150 °C). The degradation profiles reveal an expected, complex interplay between CO_2_ concentration and temperature. We propose a degradation reaction network and computationally determine the kinetics of key degradation reactions as a function of CO_2_ loading on bPEI using first-principles metadynamics simulations. These simulations show that CO_2_ exerts contrasting effects on the oxidation kinetics of bPEI: it accelerates acid-induced C–N bond cleavage while also immobilizing polymer side chains and slowing radical propagation. These findings are further confirmed by machine-learning-accelerated molecular dynamics (MD) simulations and ^1^H relaxometry experiments. The competing kinetic effects and their dependence on CO_2_ loading explain the intricate degradation profiles of bPEI under different conditions. Collectively, this work clarifies the role of CO_2_ in sorbent stability, underscores the importance of considering all atmospheric components in evaluating sorbent stability, and points to strategies for designing more durable DAC sorbents.

## Results and Discussion

### Oxidative Deactivation Profiles of bPEI/γ-Al_2_O_3_ under Different CO_2_-Air Compositions and Temperatures

The impact of CO_2_ loading on the sorbent stability as a function of temperature and CO_2_-air composition were explored using a bPEI/γ-Al_2_O_3_ sorbent (45 wt %, ∼ 100% pore fill). Sorbent deactivation experiments were conducted using thermogravimetric analysis (TGA) and ATR-FTIR spectroscopy with a flow-through cell. For the TGA, approximately 28.9 ± 0.03 mg of the model sorbent was exposed to CO_2_-free air (21% O_2_, balance N_2_) and different CO_2_/air mixtures with varying CO_2_ concentration (0.04%, 1%, and 5%) in air (21% O_2_, balance N_2_) at different temperatures. The changes in the mass of the sorbent were recorded over time. After exposure to CO_2_-free and CO_2_/air mixtures at a specific temperature, CO_2_ adsorption capacities (mmol CO_2_/g sorbent) were determined by exposing the samples to 0.04% CO_2_ in a balance He or N_2_ stream at 30 °C. Sorbent deactivation was determined by calculating the percent difference between the CO_2_ adsorption capacity of the fresh and treated (deactivated) samples. For the *in situ* ATR-IR analysis, a slurry of 45 wt % bPEI/γ-Al_2_O_3_ in methanol was added dropwise to an ATR-IR ZnSe crystal for sample analysis.

**Figure 1:**
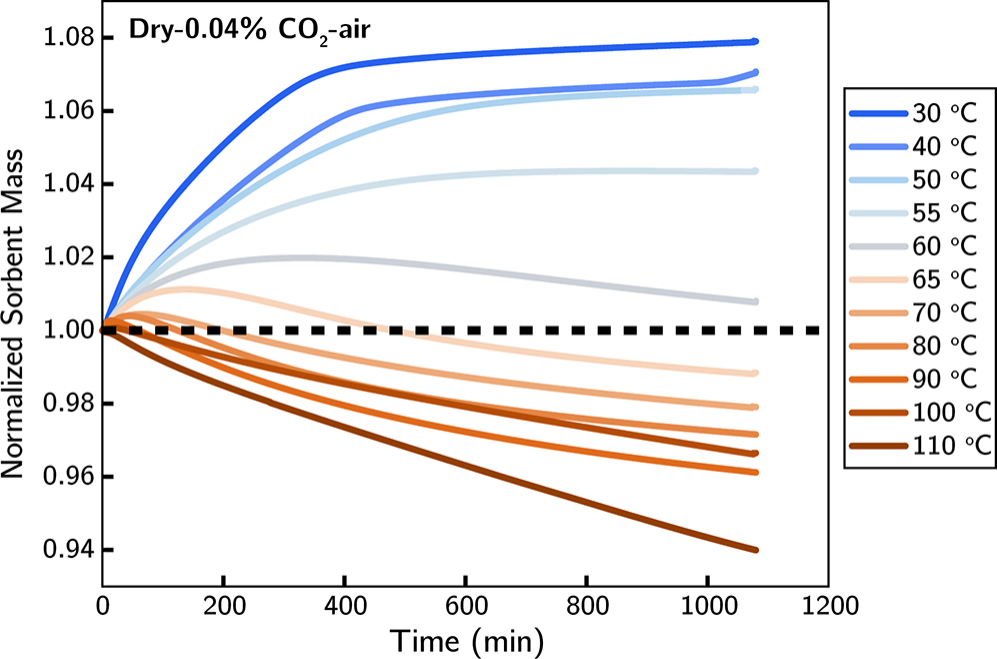
Sorbent mass change, normalized to the initial mass, under 0.04% CO_2_-air from 30–110 °C.

Figure 1 shows the sorbent mass change over time for different temperatures (30–110 °C) under the dry 0.04% CO_2_-air mixture. In the temperature range between 30 and 55 °C, a continuous increase over time in the sorbent mass was observed throughout the exposure period (18 h). As the temperature increases from 55 to 60 °C, the sorbent mass profile shows an initial mass increase followed by slight decrease starting around 330 min, at which point the curve continues to decrease gradually. This trend continues above 60 °C, with the transition from mass increase to decrease occurring faster as the temperature rises.

**Figure 2:**
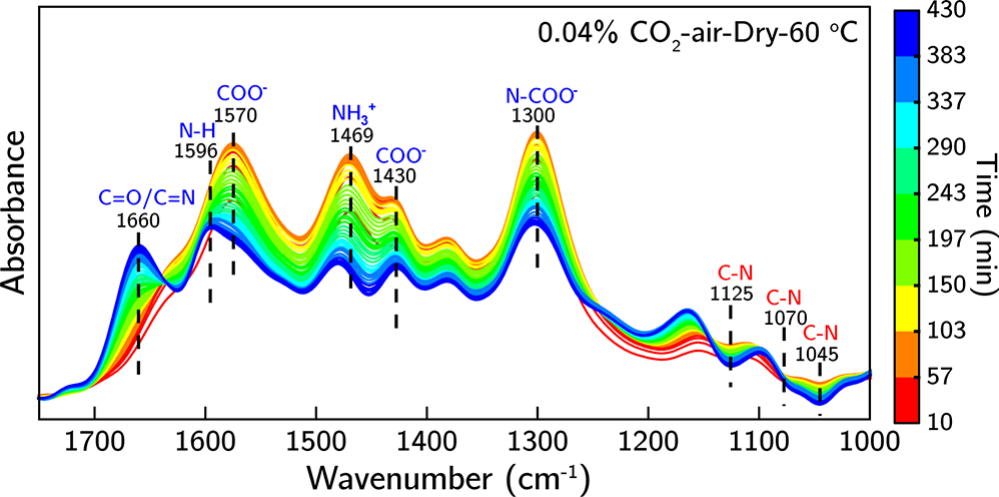
*In situ* ATR-IR spectra (1750–1000 cm^–1^) of bPEI/γ-Al_2_O_3_ sorbent deactivation under 0.04% CO_2_-air at 60 °C for 430 min.

To gain insight into the chemical speciation during oxidation, *in situ* ATR-IR experiments were conducted at different temperatures under 0.04% CO_2_-air for 18 h. The *in situ* ATR-IR spectra collected at 60 °C (Figure 2) show ammonium carbamate formation (asymmetric and symmetric COO^–^ stretching ∼1570 cm^–1^ and ∼1430 cm^–1^, NH_3_^+^ symmetric deformation ∼1463 cm^–1^, and N-COO^–^ skeletal vibration ∼1300 cm^–1^) in dominance during the early stages of the exposure (∼180 min).^32−35^ However, around 180–200 min, peaks associated with carbonyl/imine (C=O/C=N) at ∼1660 cm^–1^ appear, confirming oxidative sorbent deactivation,^21,25,36^ along with a gradual reduction in the intensity of bands associated with ammonium carbamate. Around 330 min, where the transition from sorbent mass increase to decrease occurs in Figure 1, an N–H bending band (∼1595 cm^–1^) starts to form, indicating the formation of new primary amine species as a product of the deactivation process (assigned to C–N bond cleavage events at secondary amines).^21,25^ Similar to the C=O/C=N bands, the N–H bending band continues to increase in intensity, suggesting the continuous formation of new primary amine species and sorbent deactivation.

**Figure 3:**
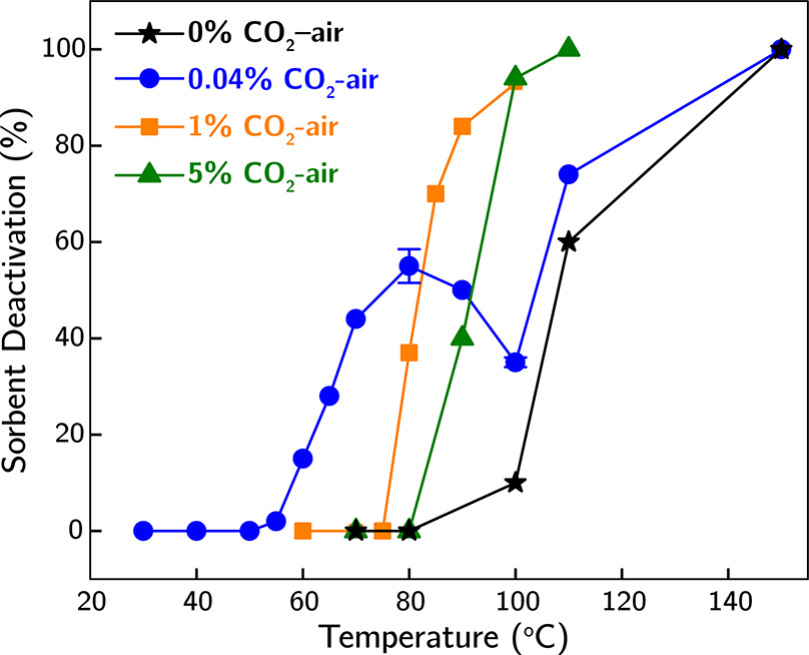
Sorbent deactivation (loss in CO_2_ adsorption capacity) under dry 0.04%, 1%, and 5% CO_2_-air for 18 h as a function of temperature. The error bars for 0.04% CO_2_-air at 80 and 100 °C indicate the standard deviation of deactivation based on three replicate runs of experiments.

Correspondingly, the CO_2_ adsorption capacity measurement of the sorbent after exposure to 0.04% CO_2_-air for 18 h at 60 °C, illustrated in Figure 3, shows noticeable sorbent deactivation (15%) at 60 °C from the exposure. The significant deactivation at 60 °C, the continuous formation of carbonyl/imine species beyond 200 min, the simultaneous occurrence of the transition from sorbent mass increase to decrease, and the formation of new primary amine species imply that the sorbent mass trend (increase followed by decrease) shown in Figure 1 indicates the transition from CO_2_ adsorption to sorbent deactivation.

In comparison, *in siu* ATR-IR spectra collected at 55 °C (Figure S1) show no noticeable C=O/C=N stretching (∼1660 cm^–1^) or N–H bending (∼1595 cm^–1^) bands, with ammonium carbamate species dominant throughout the 18-h exposure. Similarly, Figure 3 shows no significant deactivation (∼2%, compared to 15% deactivation at 60 °C) after exposure to 0.04% CO_2_-air for 18 h at 55 °C. The dominance of carbamate species throughout the exposure period and the negligible sorbent deactivation indicates that the continuous increase in mass observed in Figure 1 at 55 °C is due to net CO_2_ adsorption on to the sorbent from the CO_2_-air mixture under those conditions.

In general, the presence of CO_2_ in the gas mixture, up to 5% (the highest concentration tested here), accelerates sorbent deactivation compared to CO_2_-free conditions, regardless of temperature, as shown in Figure 3. Figures S2 and S3 illustrate faster CO_2_ uptake under 1% and 5% CO_2_-air compared to 0.04% CO_2_-air, with similar transitions from CO_2_ adsorption to sorbent deactivation. However, this transition occurs at higher temperatures under more concentrated CO_2_ conditions. Similarly, Figure 3 shows higher onset temperatures for sorbent deactivation under 1% and 5% CO_2_-air compared to 0.04% CO_2_-air. Furthermore, the extent of deactivation is consistently higher under 0.04% CO_2_-air compared to other gas mixture compositions below 80 °C. Collectively, these findings indicate increased sorbent stability when exposed to more concentrated CO_2_ in the low to intermediate temperature range, hinting at the potential effect of high-concentration CO_2_ on amine protection under certain conditions as observed by Heydari-Gorji and Sayari.^20^

The extent of sorbent deactivation generally increases monotonically with temperature upon exposure to most gas mixtures. Unexpectedly, when the sorbent was exposed to the 0.04% CO_2_-air mixture, deactivation depends nonmonotonically on temperature with a local maximum and minimum observed at 80 and 100 °C, respectively. Consequently, the difference in sorbent deactivation between the 0.04% CO_2_-air mixture and the CO_2_-free air mixture narrows with increasing temperature, beginning at 80 °C. This unique behavior associated with 0.04% CO_2_-air also leads to a lower extent of sorbent deactivation at temperatures above 90 °C compared to 1% CO_2_-air, and above 100 °C compared to 5% CO_2_-air.

### Proposed Oxidation Mechanism of bPEI

**Scheme 1:**
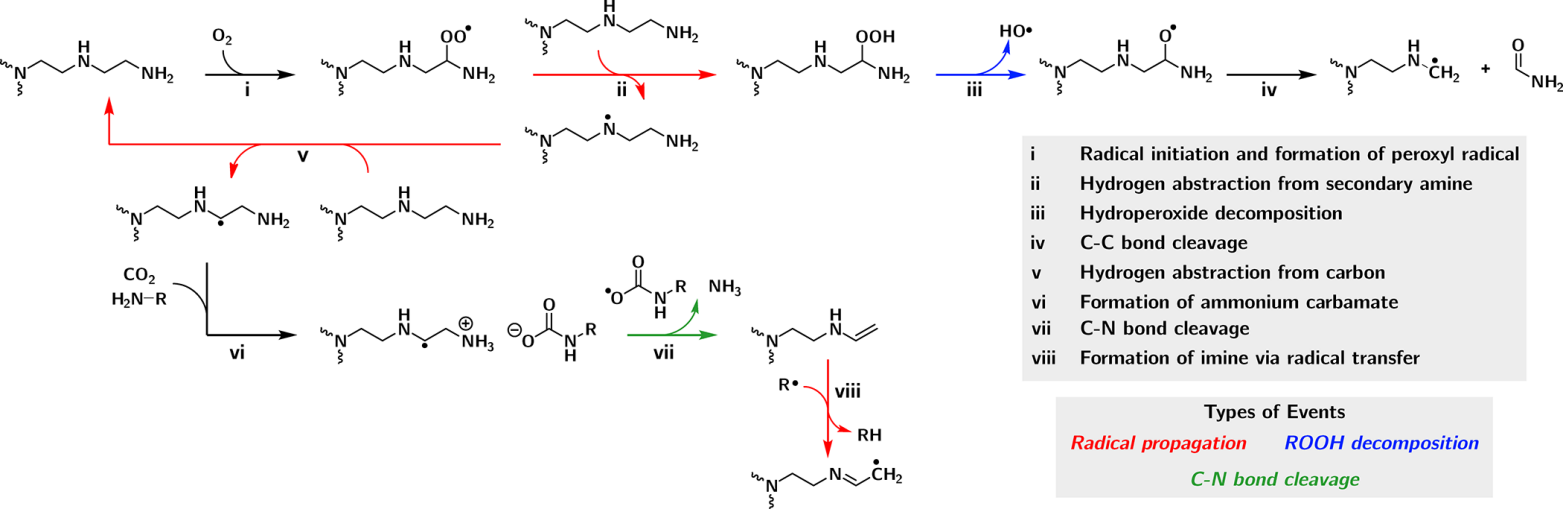
Proposed Reaction Network for bPEI Oxidation This diagram illustrates the oxidation process of a side chain, for visual clarity, in branched poly(ethylenimine) (bPEI). The depicted reactions are representative and can be analogously applied to other sites within the bPEI structure.

To elucidate the mechanisms underlying the observed CO_2_-dependent kinetic behavior, we first developed mechanistic postulates of bPEI oxidative degradation. Building upon the general understanding of polymer oxidation,^37−40^ and unique chemistry of amines, as identified in our prior investigations,^16,25,41,42^ we propose pathways for the formation of major degradation products–specifically amides and imines–on bPEI. Scheme 1 outlines a comprehensive oxidation reaction network of bPEI using a polymer side chain containing both primary and secondary amines, as a representative exemplar. Several examples of realistic bPEI molecules are shown in Scheme S1.

The oxidation reaction network starts with a common radical initiation step, which can be instigated by reactive oxygen species under oxidizing conditions, leading to the generation of initial alkyl radicals exhibiting a strong affinity for triplet O_2_. Subsequent binding of O_2_ molecules to these alkyl radicals produces peroxyl radicals (ROO·). These two steps were lumped together as event (i). (ROO·) serves as one of the major drivers for radical propagation via hydrogen abstraction reactions. This process yields alkyl hydroperoxide (ROOH) intermediates, subsequently decomposing into hydroxyl (HO·) and alkoxyl radicals (RO·), ultimately leading to the formation of amide products via nearly barrierless C–C bond cleavage that is further elaborated below in a subsequent section. This pathway, encompassing events (i) to (iv) as illustrated in Scheme 1, has been previously investigated for a range of aminooligomers,^42^ revealing the critical role of ROOH decomposition in modulating the overall oxidation kinetics.

In the presence of alkyl radicals, alternative pathways involving C–N bond cleavage become pertinent, particularly when chemisorbed CO_2_, in the form of a carbamic acid or carbamate/ammonium pair, is in close proximity with the alkyl radical. Events (v) to (viii) depict, as an example, a pathway of CO_2_-catalyzed C–N cleavage, featuring two primary amines as the CO_2_ adsorption site and the proton acceptor. This pathway necessitates two radical propagation reactions, (v) and (viii), and is expected to result in the formation of volatile NH_3_ and an imine moiety on the bPEI side chain.^25,41^

### Kinetic Dependence of Key Oxidation-Driving Reactions on CO_2_ and Their Underlying Chemistry

As described, the proposed reaction network comprises three primary categories of events: ROOH decomposition, C–N bond cleavage, and radical propagation. ROOH decomposition and C–N bond cleavage are known to exhibit moderate kinetic barriers and thus can potentially act as rate-limiting steps under certain conditions.^25,41−43^ Radical propagation, being ubiquitous and varied, serves as a prerequisite for the other two reaction categories. Its kinetics, if sluggish, could directly influence the effective degradation rates. The significant influence of CO_2_ concentration on bPEI oxidation prompts us to investigate the potential impact of CO_2_ concentration, oxidation temperature, and the resulting CO_2_ loading on bPEI on the kinetics of these key reactions.

**Figure 4:**
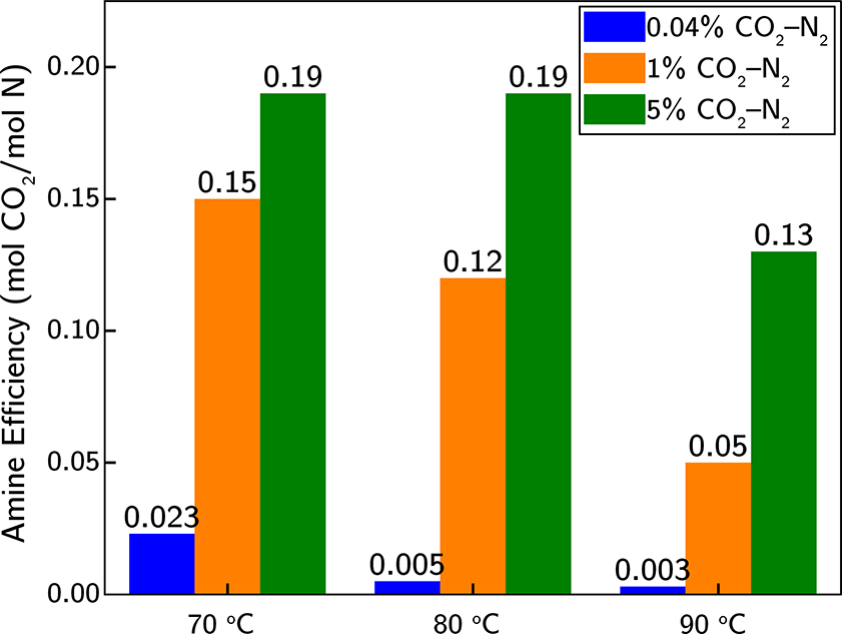
Amine efficiency at 70, 80, and 90 °C after exposure to dry 0.04%, 1%, and 5% CO_2_ -N_2_.

To determine the typical range of CO_2_ loading in fresh bPEI under the conditions used in our degradation experiments, we exposed fresh samples to gas mixtures containing 0.04%, 1%, and 5% CO_2_ balanced by N_2_ (O_2_-free) for 18 h at temperatures of 70, 80, and 90 °C. No significant mass loss or measurable sorbent deactivation were observed after these treatments, as shown in Figure S4. Through a combination of desorption experiments and elemental analyses, we determined the amine efficiencies of these samples after gas exposure. As illustrated in Figure 4, these conditions resulted in amine efficiencies ranging from 0.003 to 0.19. Notably, at all temperatures, the CO_2_ loading was significantly higher for the 1% and 5% CO_2_-N_2_ mixtures compared to the 0.04% CO_2_-N_2_ mixture.

Based on the determined range of CO_2_ loading, we set up four bPEI simulation supercells with varying CO_2_ loading for first-principles simulations to gain mechanistic insights into the oxidative degradation of bPEI in the presence of CO_2_. Each cell contains two bPEI molecules, each a molecular weight of approximately 800 Da as shown in Scheme S1. We affixed varying numbers of CO_2_ molecules randomly to primary amine sites to achieve amine efficiencies of 0, 0.03, 0.15, and 0.2 mol CO_2_/mol N. The dimensions of each cubic cell were adjusted to match the experimental bulk density of bPEI at 1.05 g/mL. Subsequently, 30 ps of AIMD simulations at 70 °C were carried out to thermally equilibrate the cells. Equilibrated structures are shown in Figure S5.

From these equilibrated cells, we conducted ab initio metadynamics simulations to determine the kinetics of ROOH decomposition reactions as a function of amine efficiency. In each cell, we replaced a hydrogen atom with an –OOH ligand on the α carbon adjacent to the primary amine site on the same bPEI side chain. For cells with CO_2_, this primary amine is one of the adsorption sites occupied by CO_2_. Following an additional 5 ps of equilibration for each cell, we initiated metadynamics to determine the barriers of ROOH decomposition. The core concept of metadynamics involves augmenting the natural DFT potential energy surfaces with a sequence of Gaussian-shaped biasing potentials that build up during the AIMD trajectory. By adding these biasing potentials as minor perturbations throughout the dynamics trajectory, the system can be gradually guided from one equilibrium state to another area of interest in the configurational space. The collection of biasing potentials accumulated over the course of the simulation reports on the underlying free energy surface, including both the enthalpic and entropic parts of the reaction.

**Figure 5:**
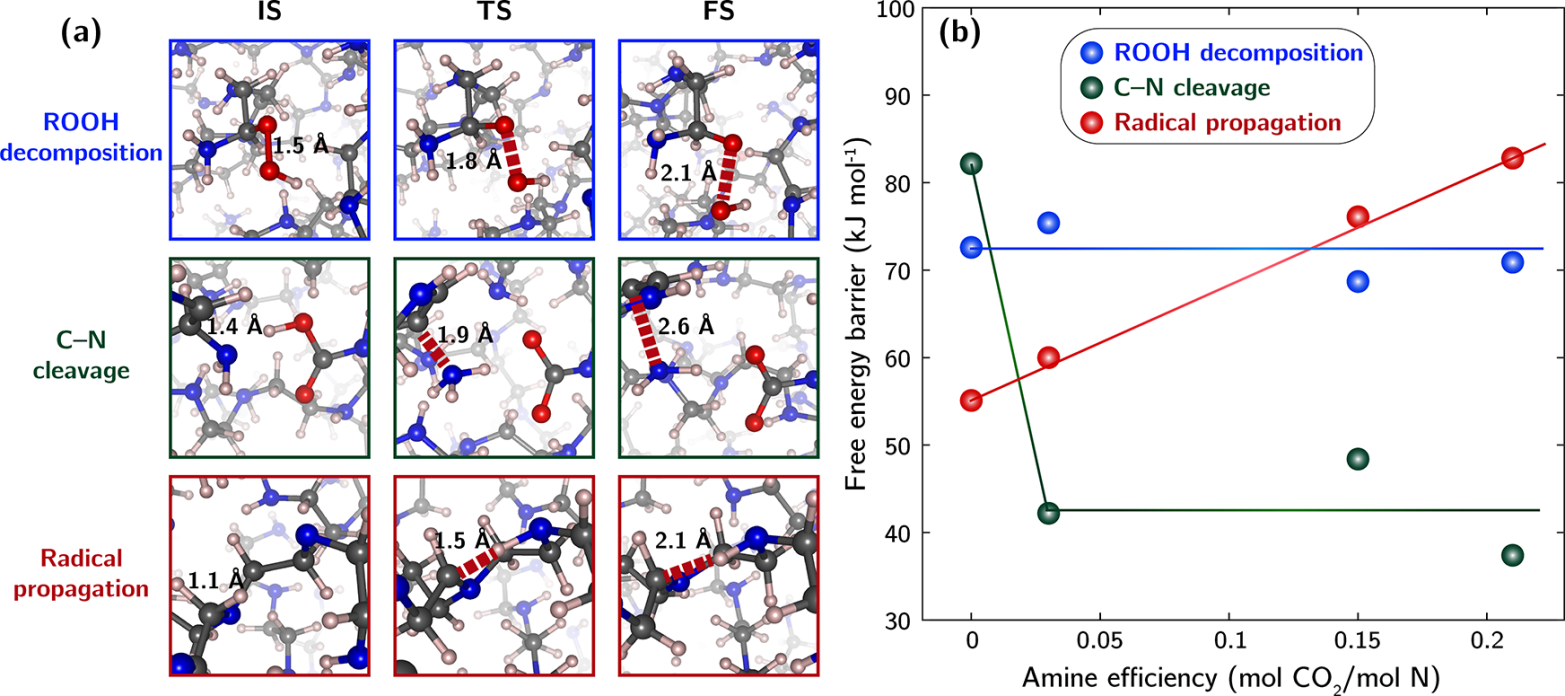
(a) Structural snapshots from metadynamics simulations showing the initial (IS), near-transition (TS), and final states (FS) for typical reactions on bPEI: ROOH decomposition, C–N bond cleavage, and aminyl radical-initiated radical propagation. (b) Free energy barriers for these reactions at varying CO_2_ loadings, characterized by amine efficiency, as determined from metadynamics simulations at 70 °C. Color code for atoms: Gray–C, pink–H, red–O, blue–N.

As depicted in the first row of Figure 5(a), ROOH decomposition involves the rupture of the O–O bond into HO· and RO·, prompting the use of the O–O coordination number as the collective variable to drive the reaction. Figure S6 illustrates that the deposited biasing potentials effectively broke the O–O bond, allowing us to determine the underlying free energy barrier. As shown in Figure 5(b), the free energy barriers of ROOH decomposition hover around 70 kJ/mol and show minimal variation with amine efficiency, suggesting that proximal CO_2_ has little influence on the reaction kinetics.

The resulting HO· is anticipated to attack alkyl or aminyl groups, initiating new radicals on bPEI. To explore the fate of the other decomposition product, RO·, we conducted supplementary AIMD simulations starting from RO· intermediates. Interestingly, as depicted in Figure S7, we observed spontaneous C–C bond cleavage, leading to the formation of amides and alkyl radicals within brief AIMD simulations at room temperature. This observation suggests that RO· on bPEI is unstable and undergoes further decomposition, underscoring the mechanistic connection between ROOH decomposition and amide formation proposed in Scheme 1.

Next, we explored the effect of CO_2_ on C–N cleavage reactions. As described in Scheme 1, the process of breaking a C–N bond involves an alkyl radical on the same side chain, resulting from radical propagation. This allows for the creation of stable enamine intermediates and the release of volatile H_2_N· or NH_3_ upon C–N cleavage, i.e. event (vii). Detailed in Figure S8, the presence of a C=C bond significantly weakens the N–H bond strength, further promoting H-abstraction reactions from the aminyl group and resulting in imine products. Similarly, we performed metadynamics simulations to determine the free energy barriers associated with the cleavage of C–N bonds on bPEI that is binding CO_2_ with varying amine efficiencies. We modified each pre-equilibrated bPEI cell by removing a hydrogen atom from the beta carbon of a side chain to promote the kinetic cleavage of terminal C–N bonds. For CO_2_-containing bPEI cells, we altered the side chain where the primary amine site is linked to carbamic acid, as illustrated in Figure 5a. In simulations involving CO_2_-free bPEI, the C–N coordination number served as the sole collective variable. For CO_2_-containing bPEI simulations, we also incorporated the N–H coordination number as an additional collective variable. This allowed the systems to explore both the protonated and deprotonated states of the affected amine sites. As shown in Figure 5b, the presence of CO_2_ markedly reduces the free energy barrier associated with C–N bond rupture, almost halving it compared to the CO_2_-free scenario. However, the degree of barrier reduction does not increase further with higher CO_2_ loading, indicating that the enhanced kinetics likely result from local and short-range acid–base interactions between amines and CO_2_ and are unaffected by the global CO_2_ loading on bPEI.

**Figure 6:**
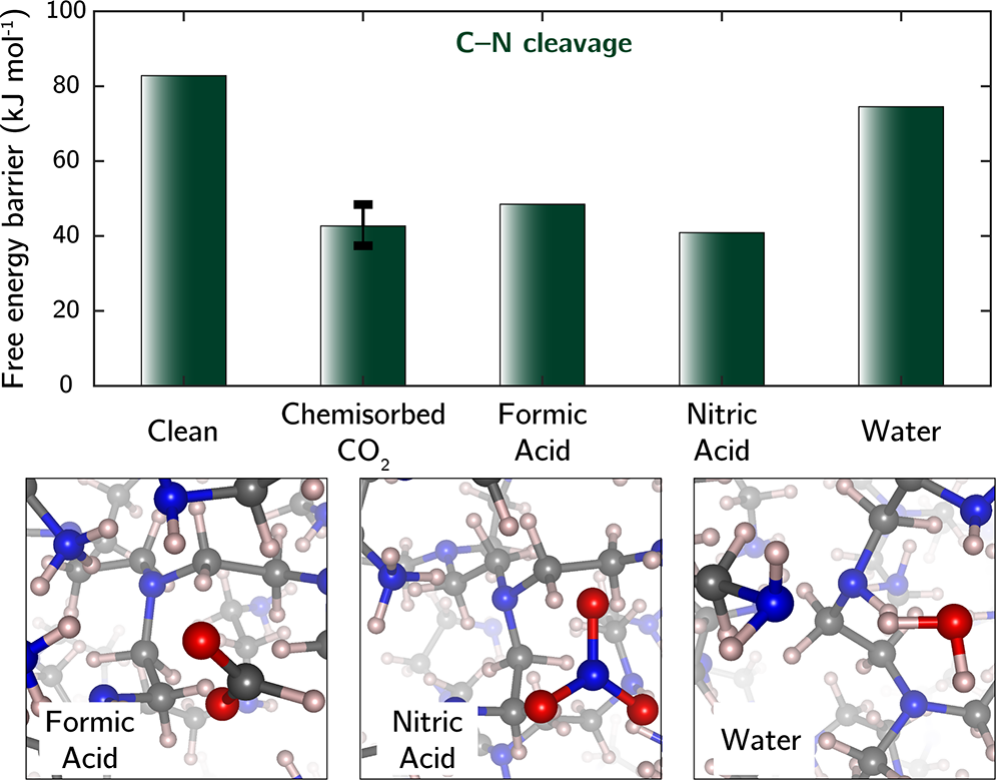
Free energy barriers for direct C–N cleavage and C–N cleavage in the presence of chemisorbed CO_2_, molecular formic acid, nitric acid, or water, as determined from metadynamics simulations at 70 °C. The vertical range for chemisorbed CO_2_ corresponds to barriers at varying CO_2_ loadings shown in Figure 5. (b). Initial structures of bPEI with molecular formic acid, nitric acid, or water are displayed at the bottom. Color code for atoms: Gray–C, pink–H, red–O, blue–N.

To confirm that C–N cleavage can be acid-catalyzed, we further explored the kinetic effects of other acids on this reaction. We built additional structural models by inserting molecular formic acid or nitric acid into the CO_2_-free bPEI cell near a primary amine site. Proton transfer from the acid to the amine was observed within 5 ps of equilibration in AIMD simulations, forming ammonium/formate and ammonium/nitrate ion pairs, as shown in Figure 6. Metadynamics revealed that the free energy barriers for C–N cleavage with these acids present were effectively reduced, lying between 40 and 50 kJ/mol, similar to the CO_2_-containing scenarios. The extent of kinetic enhancement appears to be minimally influenced by acid strength, despite nitric acid being theoretically much more acidic than formic acid and carbamic acid. As a control, we simulated the same reaction with the presence of molecular water which has a lower p*K*_a_ than amines but is theoretically neutral. The water molecule did not undergo proton transfer. Instead, it served as a hydrogen bond donor to the primary amine. This interaction lowered the free energy barrier by 8 kJ/mol compared to the neat bPEI, indicating a small kinetic promoting effect, broadly aligned with a previous report of humidity accelerating bPEI oxidation.^21^ However, the effect from water is notably weaker than those with acids involved. These findings confirm that chemical species capable of donating protons to amines, even weak acids, can indeed significantly promote the C–N cleavage reaction on bPEI.

We next investigated the potential impact of CO_2_ loading on the kinetics of radical propagation reactions. Radical propagation is essential for both the decomposition of ROOH and the C–N cleavage pathways and can manifest in various forms, including attacks on polymer repeat units by reactive oxygen species generated *in situ*, such as HO· from ROOH decomposition, or through hydrogen abstraction by alkyl peroxyl radicals (ROO·) or aminyl radicals on bPEI side chains. Due to its high reactivity, HO· likely follows collision theory and readily reacts with alkyl or aminyl hydrogen atoms along its diffusion paths. However, the rate of HO· formation and its concentration are expected to be closely linked to the kinetics of ROOH decomposition.

ROO· or aminyl radicals, on the other hand, are less reactive and radical propagation reactions initiated by these organic radicals often exhibit appreciable activation energies.^42,43^ We previously demonstrated that ROO· preferentially forms hydrogen bonds with amines in condensed-phase triethylenetetramine (TETA) and thus selectively abstracts hydrogen atoms from aminyl groups, resulting in aminyl radicals.^42^ In this study, we conducted metadynamics simulations for bPEI using the O–H coordination number as the collective variable, enabling interchain hydrogen abstraction by ROO· from any hydrogen donors. Similar to TETA, our observations, shown in Figure S9, indicate that ROO· on bPEI consistently abstracts hydrogen from secondary amines, irrespective of CO_2_ loading. The free energy barrier without CO_2_ is relatively low but significantly increases when CO_2_ is chemisorbed on the same bPEI side chain, with minimal changes observed with additional CO_2_ chemisorbed on other side chains.

In contrast, aminyl radicals do not favor forming hydrogen bonds with any surrounding hydrogen atoms. Employing the N–H coordination number as the collective variable, metadynamics simulations reveal that aminyl radicals on bPEI tend to preferentially abstract hydrogen from alkyl groups, resulting in new alkyl radicals. The preferential target for hydrogen abstraction by aminyl radicals is likely due to the relatively weaker bond strength of C–H compared to N–H on PEI as determined by a machine-learning derived bond dissociation enthalpy tool.^44^ These reactions are thus crucial for directing alkyl radicals to appropriate locations before ROOH decomposition and C–N cleavage pathways proceed. As depicted in Figure 5b, the free energy barriers of radical propagation pathways initiated from aminyl radicals show a consistently positive correlation with amine efficiency and are generally higher than those initiated from ROO·.

**Figure 7:**
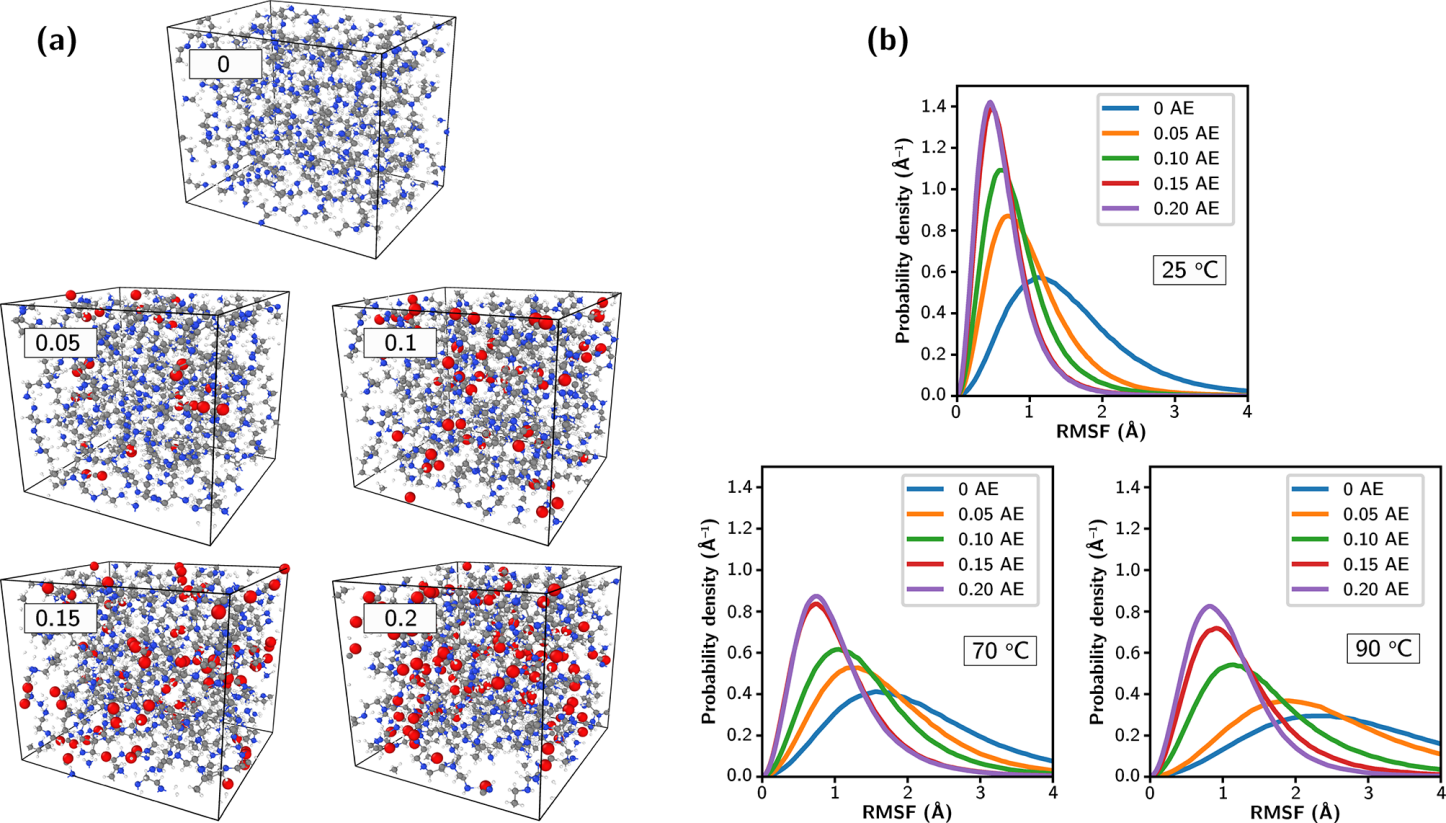
(a) Supercells of bPEI, with amine efficiency (AE) ranging from 0 to 0.2, equilibrated using MACE MD simulations. (b) Probability distribution of root-mean-square fluctuation (RMSF) of carbon and nitrogen atoms on bPEI with varying amine efficiencies at different temperatures, each based on a 2 ns trajectory of MACE MD simulations. Color code for atoms: Gray–C, pink–H, red–O, blue–N.

Higher free energy barriers for radical propagation in the presence of CO_2_ can result from the potential reduction in bPEI side chain mobility upon CO_2_ chemisorption, as decreased mobility can limit local fluctuations of side chains and increase the barrier for them to approach each other and transfer hydrogen. As previously hypothesized based on studies with epoxide-functionalized bPEI^18,45,46^ and polyol additives,^47^ functional groups such as hydroxyls that promote robust interchain interactions can reduce the mobility of polymer side chains and slow down radical propagation reactions.^48^ The chemisorption of CO_2_ generates *in situ* carbamic acids or carbamate/ammonium complexes, which theoretically can pin the side chains through strong interchain acid–base interactions, similarly reducing their mobility. To support this hypothesis, we performed machine learning-accelerated molecular dynamics (MD) simulations to assess the mobility of bPEI as a function of CO_2_ loading and temperature. First, we developed a machine learning interatomic potential based on the MACE method.^49^ Employing a random structure generation approach that reflected the molecular weight and amine distribution of bPEI, we generated five large simulation cells of bPEI with amine efficiencies ranging from 0 to 0.2 mol CO_2_/mol N, as shown in Figure 7a. Each supercell contained 16 bPEI molecules, composed of a blend of four structural variants illustrated in Scheme S1. We conducted 2.5 ns of MD for each combination of amine efficiency and temperature, using the latter 2 ns of the trajectory to compute the root-mean-square fluctuation (RMSF) of all non-hydrogen atoms in each system. The probability density of RMSF quantifies the side chain dynamics of bPEI. Distributions with larger values of RMSF indicate more flexible polymer chains.

As illustrated in Figure 7b, both CO_2_ loading and temperature significantly influence the RMSF of bPEI. Higher temperatures generally lead to broader RMSF peaks with larger values, indicating increased bPEI mobility. In contrast, as CO_2_ loading increases, the peak center of RMSF at each temperature shifts to lower values and becomes narrower, showing that chemisorbed CO_2_ effectively reduces bPEI mobility. Overall, chemisorbed CO_2_ at higher temperature makes bPEI behave similarly to CO_2_-free bPEI at lower temperature. For instance, the RMSF line shape of CO_2_-free bPEI at 25 °C closely resembles that of bPEI loaded with 0.1 mol CO_2_/mol N at 70 °C. The difference in RMSF line shape between 0.15 and 0.2 mol CO_2_/mol N becomes subtle, especially at lower temperatures, suggesting that the impact of CO_2_ levels off at higher CO_2_ loadings.

Next, we used ^1^H relaxometry to experimentally investigate the mobility of bPEI preloaded with varying quantities of CO_2_, corresponding to amine efficiencies between 0 and 0.16 mol CO_2_/mol N. These experiments allowed us to determine the spin–lattice relaxation time (T_2_) and spin–spin relaxation time (T_2_) for each sample.^50^ T_1_ reflects large-scale motions, such as translational diffusion, with lower T_1_ values indicating higher molecular mobility. Conversely, T_2_ serves as an indicator of chain stiffness, with higher T_2_ values suggesting greater chain mobility.

**Figure 8:**
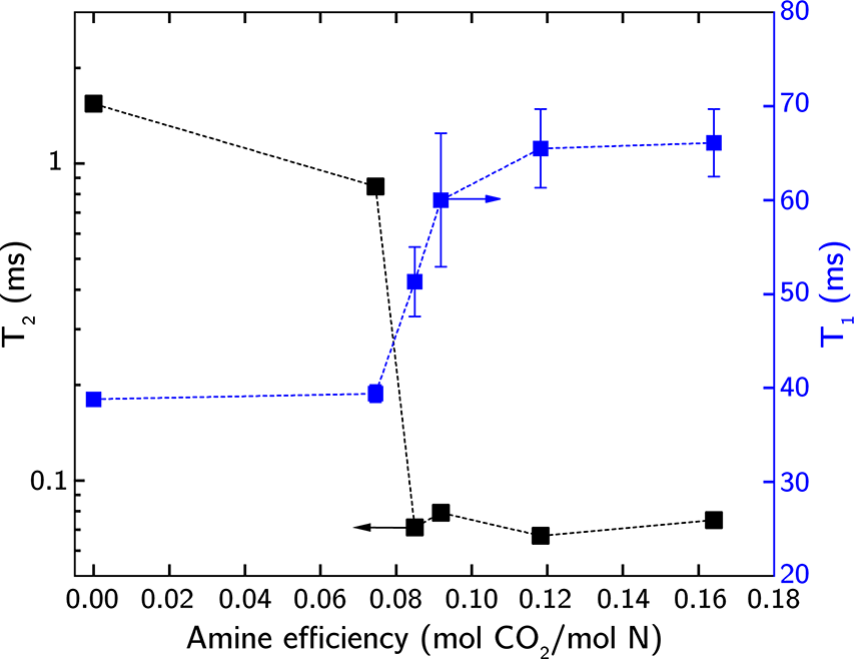
^1^H NMR relaxometry (T_1_ and T_2_) for bPEI preloaded with varying amounts of CO_2_.

Figure 8 presents the measured T_1_ and T_2_ values as a function of amine efficiency. The general trends show that both side-chain and diffusional mobility of bPEI decrease with increasing CO_2_ loading, consistent with observations from MACE MD simulations. T_2_ relaxation times decreased following the adsorption of CO_2_, with a noticeable drop between 0.075 and 0.085 mol CO_2_/mol N. Similarly, T_1_ relaxation times exhibit a rapid increase above 0.075 mol CO_2_/mol N, followed by a more gradual plateau. These trends are similar to the behavior observed in the physical^51^ and electrical^52^ properties of polymers and polymer blends experiencing a percolation effect, where the formation of a continuous phase within the polymer reaches a critical concentration. This suggests the existence of a critical threshold of CO_2_ loading for bPEI, ∼ 0.08 mol CO_2_/mol N, beyond which sufficient carbamate cross-links are formed, leading to a significant reduction in bPEI mobility detectable by ^1^H relaxometry.

### CO_2_ Loading-Dependent Kinetic Regimes of bPEI Oxidation

**Scheme 2:**
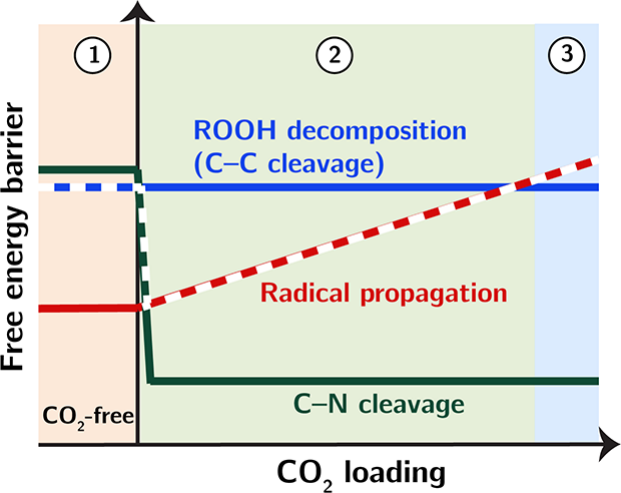
Schematic Illustration of Kinetic Dependence of Key Oxidation-Driving Reactions on CO_2_ Loading Colored boxes with indices represent different kinetic regimes. Transitions of rate-determining steps are conceptually highlighted with dashed lines.

These findings collectively depict how CO_2_ influences the oxidative degradation of bPEI, which can be characterized as different kinetic regimes determined by CO_2_ loading as shown in Scheme 2. In the absence of CO_2_ (kinetic regime ①), the kinetics of ROOH decomposition and C–N cleavage reactions are comparable, jointly limiting the overall oxidation rate. When CO_2_ is introduced to bPEI, C–N cleavage reactions are significantly catalyzed, becoming the preferred degradation pathway over ROOH decomposition. On the other hand, radical propagation reactions are decelerated by chemisorbed CO_2_, with the extent of deceleration increasing with CO_2_ loading. This shift makes radical propagation the rate-limiting step instead of ROOH decomposition and C–N cleavage. In this kinetic regime ②, CO_2_ generally promotes the oxidation kinetics of bPEI by accelerating C–N cleavage pathways, but higher concentrations of CO_2_ slow down the prerequisite radical propagation, counteracting its catalyzing effect.

The transition from kinetic regime ① to ② explains the general accelerating effect of CO_2_ on bPEI oxidation shown in Figure 3. Additionally, it reveals the mechanistic origin of higher onset temperatures associated with higher CO_2_ concentrations. As noted earlier, high CO_2_ loading does not further lower the free energy barrier of C–N cleavage reactions, but it is expected to increase the number of active sites, enhancing the overall C–N cleavage kinetics. Under the conditions of 0.04% CO_2_-air and an intermediate temperature range, CO_2_ loading is relatively low and continues to drop as temperature increases. This can result in insufficient active sites catalyzing C–N cleavage, leading to a dip in the oxidation rate, as shown in Figure 3, before ROOH decomposition pathways significantly contribute to the overall degradation kinetics. Figure S10 presents additional data manifesting the correlation between CO_2_ loading and sorbent deactivation.

Theoretically, it is possible to reach a kinetic regime (③) where radical propagation is slowed down to such an extent that the overall oxidation kinetics become slower than in the absence of CO_2_. The conditions we explored, as shown in Figure 3, and the fixed-duration oxidation protocol did not capture such a kinetic regime. Achieving this might require a significantly higher CO_2_ concentration to maintain the saturation limit of CO_2_ loading on bPEI at or above the oxidation onset temperature of bPEI exposed to dry air without CO_2_.

Indeed, Heydari-Gorji and Sayari observed that PEI-SBA-15, after 30 h of exposure to a 7.5% CO_2_/10.5% O_2_ /82% N_2_ gas mixture at 100 °C, showed a significantly lower CO_2_ uptake loss (3%) compared to the 50% loss after the same duration of exposure to a 10.5% O_2_ /89.5% N_2_ mixture.^20^ Notably, these oxidation experiments^20^ were conducted under subambient O_2_ pressure, significantly lower than the 20–21% O_2_ used in our experiments. Given that bPEI oxidation has a positive rate order with respect to O_2_,^16^ this difference can influence the relative impact of CO_2_ on overall degradation kinetics. Additionally, their use of a different oxide support for PEI, i.e. silica SBA-15, further complicates a direct comparison with our results. Nonetheless, their findings confirm the existence of a kinetic regime (③) where the protective effect of CO_2_ is more pronounced, thereby reconciling conflicting reports in the literature about the role of CO_2_ in amine oxidation.

## Conclusions

In summary, our integration of experiments and first-principles simulations reveals the complex impact of CO_2_ on the oxidative degradation kinetics of aminopolymer sorbents, notably Al_2_O_3_-supported bPEI. We find that chemisorbed CO_2_ acts as a catalyst, expediting bPEI oxidation through the acceleration of acid-induced C–N bond cleavage. Conversely, elevated CO_2_ concentrations induce extensive acid–base interactions, effectively immobilizing polymer side chains and impeding radical propagation, thereby retarding the overall oxidation kinetics. These divergent mechanisms give rise to intricate oxidation profiles, highlighting the intricate nature of CO_2_’s influence on aminopolymer stability. Our findings reconcile seemingly conflicting data in the literature on the impact of CO_2_ on amine oxidation while explaining the subtle effect of CO_2_ concentration on amine stability.

Beyond enhancing our fundamental understanding of sorbent behavior, our findings hold practical significance for the optimization of CO_2_ capture and sorbent regeneration processes, offering pathways to mitigate the environmental impact of sorbent degradation. Furthermore, our identification of high side chain mobility and acidic chemical environments as key factors in accelerating aminopolymer oxidation suggests promising avenues to reduce the oxidation rate of amine sorbents. Introducing functional groups, additives, or oxide support with appropriate surface chemistry that impede polymer side chain mobility or neutralize intrinsic or *in situ* generated acids emerges as a viable strategy to effectively counteract aminopolymer oxidation, thereby advancing the development of more resilient sorbent materials for carbon capture applications.

## Experimental Section

### Materials & Sorbent Synthesis

Branched poly(ethylenimine) (800 g/mol) was purchased from Sigma-Aldrich while a mesoporous gamma-alumina (γ-Al_2_O_3_) (Catalox HP 14/150) support was supplied by Sasol. The aminopolymer sorbent material is prepared by wet impregnating 45 wt % branched PEI onto a mesoporous γ-Al_2_O_3_ following the procedure by Pang et al.^23^ All gas mixtures (N_2_ (UHP 99.999%), ultra zero grade air (21% O_2_ balance N_2_), 400 ppm, 1%, and 5% CO_2_ balance air (21% O_2_ balance N_2_), and 400 ppm, 1% and 5% CO_2_ balance N_2_) were purchased from Airgas.

### Sorbent Characterization

After the sorbent synthesis, the total organic content and pore filling of the sorbent were determined using thermogravimetric combustion analysis and N_2_ physisorption isotherms, respectively. N_2_ physisorption isotherm experiments were conducted using Micromeritics TriStar II 3020 Version 3.02 at 77 K while organic combustion analysis was conducted using TGA 550 (TA Instruments).

For the N_2_ physisorption experiments, about 150 mg of the sorbent (bPEI/γ–Al_2_O_3_) or the support (γ-Al_2_O_3_) were used for pore volume, surface area, and pore size analysis. Before each experiment, the samples were pretreated for 10 h under vacuum at 120 °C (γ-Al_2_O_3_) and 60 °C (PEI/ γ-Al_2_O_3_). These pretreatment conditions resulted in a similar percent loss in mass as the pretreatment prior to CO_2_ adsorption (under N_2_ at a flow rate of 100 mL/min at 100 °C for 1 h) due to removal of CO_2_ and water, suggesting that the sorbent was similarly activated in both cases. The BET surface area and the BJH adsorption pore volume of the support were measured to be 133 m^2^/g and 0.82 ± 0.05 cm^3^/g, respectively. Similarly, the bPEI/γ-Al_2_O_3_ sorbent’s BET surface area and the BJH adsorption pore volume of the support were measured to be 0.68 m^2^/g and 0.03 cm^3^/g, respectively.

Organic combustion analysis was conducted by pretreating the sample at 100 °C for 1 h under N_2_ to desorb weakly adsorbed species (CO_2_ and H_2_O). Following the pretreatment, the sample was heated to 900 °C at a ramp rate of 10 °C/min under air (ultra zero grade air). The mass loss after the pretreatment was taken as the organic content of the sorbent.

The C, H, and N content of samples pre- and postdeactivation experiments were analyzed by Atlantic Microlabs. Based on the elemental analysis results, the amine efficiency of the sorbents and changes in C, H, and N content postdeactivation were determined.

### Sorbent Deactivation

The sorbent deactivation experiments were conducted using TGA-DSC (TA Instruments Q600). In a typical experiment, 28.9 ± 0.04 mg of 45 wt % bPEI/γ-Al_2_O_3_ sorbent was loaded into a 40 μL alumina ceramic pan and exposed to CO_2_-free air (21% O_2_/balance N_2_), CO_2_-air (0.04%, 1%, or 5% CO_2_ balance air) or CO_2_-N_2_ (0.04%, 1%, and 5% CO_2_ balance N_2_) mixtures at different temperatures (30–150 °C) and constant pressure (1 atm). Before each experiment, the samples were pretreated under N_2_ at a flow rate of 100 mL/min at 100 °C for 1 h. During the pretreatment, the desorbed CO_2_ and H_2_O contents were monitored using a LI-COR 850 H_2_O/CO_2_ analyzer.

Following the pretreatment, the sample was heated to the desired temperature set point at a ramp rate of 5 °C/min. Once the temperature equilibrated at the desired temperature, the flow was switched to the desired gas mixture (CO_2_-free air, 21% O_2_ balance N_2_, CO_2_-air (0.04%, 1%, or 5% CO_2_ balance air) or CO_2_-N_2_ (0.04%, 1%, and 5% CO_2_ balance N_2_)) at 100 mL/min and held isothermal for 18 h. After exposure for 18 h, the flow was switched back to N_2_, and the temperature was cooled to 25 °C at a ramp rate of 20 °C/min.

### CO_2_ Adsorption Experiments

Following the deactivation experiments, CO_2_ adsorption measurements were conducted to evaluate the impact of the deactivation by the change in the CO_2_ adsorption capacity of the sorbent pre- and postdeactivation experiments. A TGA (TA Instruments Q500) was used for the CO_2_ adsorption capacity measurements.

For each experiment, ∼ 22 mg sorbent was loaded onto a 50 μL platinum pan and pretreated at 100 °C under He or N_2_ gas at a flow rate of 90 mL/min for 1 h. After the pretreatment, the temperature was cooled at a ramp rate of 10 °C/min to 30 °C under He or N_2_ gas.

Once the temperature equilibrated at 30 °C, CO_2_ adsorption measurements were conducted by exposing the sorbent to 400 ppm of CO_2_ (balance He or N_2_) at 90 mL/min for 3 h. Next, sorbent regeneration was conducted by heating the sorbent to 100 °C at a ramp rate of 10 °C/min and holding isothermal at 100 °C for 1 h under He or N_2_ at 90 mL/min. After achieving complete CO_2_ desorption in 1 h, the temperature was cooled to 30 °C at a ramp rate of 10 °C/min.

### *In Situ* ATR-IR Spectroscopy

*In situ* IR spectra were collected using a Thermo Scientific Nicolet 8700 FTIR spectrometer. A HATR-IR (horizontal attenuated total reflection infrared) heated flow-through cell equipped with a ZnSe crystal was employed to observe molecular level changes and monitor the formation of new functional groups in the bPEI/γ-Al_2_O_3_ sorbent due to the exposure to the different gas mixtures at varying temperatures. Before every experiment, 45 wt % PEI was dissolved in 10 mL methanol by stirring in a 20 mL vial at room temperature (∼21 °C). Once the PEI/methanol solution was stirred for about 2 min, the desired amount of γ-Al_2_O_3_ was added. The bPEI/γ-Al_2_O_3_/methanol mixture was stirred overnight at room temperature (∼21 °C) in a 20 mL vial to prepare a slurry mixture. After stirring overnight, the desired amount of slurry was placed in an open 20 mL vial in a glass desiccator under vacuum for about 5 min to remove some of the methanol. Immediately after the removal of some methanol, the bPEI/γ-Al_2_O_3_ /methanol slurry was added dropwise to the ZnSe crystal. Once deposited on the ZnSe crystal, the slurry was heated to 100 °C from room temperature (∼21 °C) for 1 h under N_2_ at 100 mL/min to remove the remaining methanol and other weakly sorbed species. IR peaks indicating the presence of methanol (such as O–H stretching band ∼3500 cm^–1^ and C–O stretching ∼1070 cm^–1^)^53^ in the slurry at room temperature (∼21 °C) gradually disappeared during the heating to 100 °C. Figure S11 shows the gradual disappearance of the methanol.

A ∼ 10 μm bPEI/γ-Al_2_O_3_ film formed after the pretreatment. SEM imaging was used to measure the thickness of the film. Following the pretreatment, the temperature was set to the desired temperature under N_2_ at 100 mL/min. Once the temperature stabilized, the flow was switched to the desired gas mixture: CO_2_-free air (21% O_2_/balance N_2_), CO_2_-air (0.04%, 1%, or 5% CO_2_ balance air) or CO_2_-N_2_ (0.04%, 1%, and 5% CO_2_ balance N_2_) mixtures at 100 mL/min and the temperature was held isothermal for 18 h. During the exposure to the deactivating mixtures, sample spectra were collected for the entire 18 h using Thermo Scientific Omnic software.

### Controlling Amine Efficiencies of PEI/gamma–Al_2_O_3_ Sorbent

Samples with controlled amine efficiencies were prepared using a custom-built breakthrough curve analyzer. Approximately 200 mg of bPEI/γ-Al_2_O_3_ was loaded into a 1/4″stainless steel column with on–off valves placed at either end. Following degassing under N_2_ flow at 100 °C overnight, the sample was exposed to CO_2_ at concentrations between 0 and 400 ppm in N_2_ at 30 °C for up to 3 days. The CO_2_ concentration was monitored using an LI-830 infrared CO_2_ sensor (LICOR, USA), with the resulting breakthrough curve being used to determine the CO_2_ capacity of the composite (Supplementary Figure S12, Table S1). Amine efficiencies, in mol CO_2_/mol N, were calculated by dividing adsorption capacities by the nitrogen content of the sample, measured via elemental analysis (Midwest Microlab, USA). Following adsorption, samples were isolated and transferred to glass vials inside of a glovebox purged with Argon. This step was necessary to avoid the adsorption of additional CO_2_ or water vapor from the atmosphere, both of which have been found to impact ^1^H relaxation.^54−56^

### ^1^H Relaxometry

^1^H T_1_ and T_2_ NMR relaxometry measurements were carried out with a single-sided PM2 NMR-MOUSE (Mobile Universal Surface Explorer) (Magritek, GmbH) operating at a frequency of 28.05 MHz for ^1^H T_1_ with the static magnetic field gradient of 39.9 T/m. All measurements were collected using a Kea2 spectrometer and Prospa software. Signals were detected by a horizontal slice detection area of 12.5 × 12.5 mm^2^ configured to the maximum penetration depth of 1.9 mm. For these measurements, a slice thickness of ∼115 μm (acquisition time of 5 μs) was used to reduce echo time and maximize the acquisition volume of signal. A radio frequency pulse length of 1.8 μs with varying amplitudes was used for the 90° and 180° pulses in the Carr–Purcell–Meiboom–Gill (CPMG) and T_1_ saturation recovery experiments for measuring T_2_ and T_1_, respectively. CPMG experiments used 500 echoes with an echo time of 24 μs, and between 512 to 2048 acquisition scans to improve signal-to-noise. For T_1_ saturation recovery experiments the recovery delay was incremented exponentially with 24 steps between 0 to 1200 ms max recovery time, and a CPMG detection was used for detecting each T_1_ recovery increment using the same echo time of 24 μs, coadding the first 10 echoes and acquiring 512 scans to improve signal-to-noise. Measurements were conducted at room temperature measured as the ambient temperature of the space around the stage of the MOUSE, ∼ 22 °C. A note that in this work we refer to T_2_ values but due to the inhomogeneous field inherent to the NMR-MOUSE, the measured T_2_ relaxation time is actually an effective T_2_ relaxation (T_2,eff_) because of off-resonance effects.

### Ab Initio Molecular Dynamics and Metadynamics

NVT ab initio molecular dynamics (AIMD) were performed for conformational sampling of flexible bPEI structures with the Vienna ab initio simulation package (VASP), version 5.4.4,^57^ using the projector augmented wave treatment of core–valence interactions^58,59^ with the Perdew–Burke–Ernzerhof (PBE) generalized gradient approximation for the exchange correlation energy.^60^ The energy cutoff for the plane-wave basis set was set to 400 eV and the Brillouin zone sampled with a Γ-point only. The self-consistent-field electronic energies were converged to 10^–4^ eV. The time step was set to 1 fs, and the Nose-Hoover thermostat was used to maintain the temperature at 473 K. Cubic cells containing two bPEI molecules were used in these simulations. Cell length was set to reproduce a bPEI density of 1.05 g/mL in each cell.

To compute the free energy barrier of elementary reactions at finite temperatures, metadynamics were performed. Metadynamics is a nonequilibrium molecular dynamics method capable of efficiently sampling free energy surfaces of complex reactions.^61^ We selected coordination number (CN) as the collective variable (CV), previously shown to be effective in promoting rare reaction events involving bond breaking and/or formation^62^ and mathematically defined as
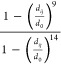
1where *d*_ij_ is the actual distance between atom i and j, and *d*_0_ is the reference distance as the boundary of being bonded or not between the two atoms.

For ROOH decomposition reactions, the CN between the two O on the hydroperoxide was used as the CV. *d*_ij_ was set to 2 Å for the CN. For H-abstraction reactions starting from O_ROO·_, the CV was set to be the sum of CNs between O_ROO·_ and all H atoms on every side chain except for the one where ROO· is located to purposefully capture interchain events. For H-abstraction reactions starting from alkyl aminyl radicals, the CV was set to be the sum of CNs between N_aminyl radical_ and all H atoms on every side chains except for the one where the aminyl radical is located. *d*_ij_ for all H abstraction reactions was set to 1.1 Å. In this way, metadynamics would be able to identify the preferred donor H atom without bias. For the C–N cleavage reaction on clean bPEI without CO_2_, the CN between N and C next to a preexisting alkyl radical is used as the CV. *d*_ij_ was set to 2. For C–N cleavage reactions in the presence of CO_2_, two CVs were used: the CN between N and C next to a preexisting alkyl radical, and the CN between N of the affected amine and all H associated with amines and the carbamic acid. *d*_ij_ was set to 2 and 1.1 Å, respectively. For simulations with 1 CV, the height of each biasing Gaussian potential was set to 0.0025 eV and the width 0.02. For simulations with 2 CVs, the height of each biasing Gaussian potential was set to 0.005 eV and the width 0.04. For all simulations, biasing potentials were added every 20 MD time steps, i.e. 20 fs. This approach ensures that perturbations from metadynamics to the underlying potential energy surfaces are small enough to obtain accurate free energy barriers associated with the reactions of interest.

Following a previously reported protocol,^62^ we terminated a run of metadynamics simulation after the first barrier crossing from the reactant basin into the target product basin and computed the free energy barrier by summing up the amount of bias potentials accumulated in the reactant basin.

### Random Structure Generation

Initial simulation cells for deep potential molecular dynamics simulations were built by randomly placing 16 amine molecules (4 each of the bPEI molecules shown in Scheme S1) within a 3D-periodic cubic supercell using a Monte Carlo algorithm.^63^ A low starting density (0.1 g/cm^3^) was chosen to ensure that no overlap occurs between neighboring molecules and to make room for subsequent insertion of CO_2_ molecules. The system contained a total of 284 amine groups of which 112 were primary. The primary N atoms were treated as potential sites for CO_2_ adsorption. To create a 5% CO_2_-loaded structure, 14 primary N atoms (i.e., 5% of 284) were randomly selected out of the 112 primary N atoms and a H atom of the corresponding –NH_2_ group replaced by a carbamic acid (−COOH) group. The 10%, 15%, and 20% CO_2_-loaded structures were similarly created by attaching an appropriate number of carbamic acid groups on randomly chosen primary amine sites. All initial supercells (i.e., the original CO_2_-free structure and the structures corresponding to four different CO_2_ loadings) were then compressed to near equilibrium density (∼1 g/cm^3^) using 100 ps long NPT simulations employing the Andersen thermostat and barostat,^64^ which preserves the (cubic) cell shape during volume change. The interatomic interactions were described by the class II force field COMPASS^65^ that has been widely validated for condensed systems like polymer melts and organic liquids, including amine solvent systems.^66,67^ Long-range coulomb interactions were treated with the Ewald summation technique.^68,69^ The structures resulting from the above procedure were used as starting points for further equilibration by machine-learned (ML) force fields, as described below.

### Machine Learning Accelerated Molecular Dynamics

A machine learning interatomic potential (MLIP) for organic amines and their interaction with CO_2_ was developed using the MACE method.^49^ MACE describes the potential energy surface of the system using a graph neural network (GNN) with high-order many body message passing. In this work, the input to the GNN consists of the chemical environment within 5 Å cutoff from each atom. The GNN is trained on data containing condensed phase structures of a range of molecules ranging from ammonia to bPEI. Those molecules are NH_3_, Me–NH_2_, (Me)_2_–NH, (Me)_3_–N, triethylenetetramine (TETA), tripropylenetetramine (TPTA), tris(2-aminoethyl)amine (TAEA), branched PEI (bPEI) and their interaction with CO_2_. For bPEI, the data contained up to 26% CO_2_/N mole fraction.

Training data was self-consistently constructed using reinforcement learning. In this scheme, training data is collected on-the-fly based on the force uncertainty prediction of MLIP. More specifically, molecular dynamics simulations using the MLIP explores a vast number of atomic configurations but only those with a force error threshold above 0.1 eV/Å are selected, recomputed with density functional theory (DFT) and appended to the training data. This process is repeated until the error in atomic force prediction falls consistently below the 0.1 eV/Å threshold. Atomic configurations were sampled with molecular dynamics simulations using a Nosé–Hoover thermostat at temperatures ranging from 300 to 400 K and pressure controlled from 1 to 2000 bar.

Atomic forces, potential energies and the stress tensor of atomic structures in the MLIP training data were computed with the SCAN functional^70^ as implemented in the Quantum ESPRESSO package.^71^ Wave functions and charge density were planewave-expanded with an energy cutoff of 200 and 800 Ry, respectively. Norm conserving pseudopotentials of Troullier-Martins^72^ type replaced explicit core–valence electron interactions. Molecular dynamics simulations were performed with the MACE calculator implemented in the Atomic Structure Environment package (ASE).^73^ Temperature was controlled using the Nosé–Hoover thermostat,^74,75^ while pressure was kept constant using the Parrinello–Rahman barostat.^76^

Figures S13, S14, and S15 show that molecular dynamics simulations based on MLIP and SCAN functional produce nearly identical pair correlation functions for the 2-bPEI supercells with varying number of chemisorbed CO_2_, further confirming the robustness of the MLP and the MACE calculator. All production runs of MD simulations were performed on periodically repeating systems with more than 2200 atoms per unit cell. The compositional inhomogeneity of bPEI was modeled by a mixture of 4 molecular structures with similar molecular weight and same ratio of primary, secondary and tertiary amines. The classical equations of motion were numerically integrated with a time step of 0.5 fs. Hydrogen was replaced by deuterium, allowing longer timesteps without affecting the equilibrium statistics of the classical system. All trajectories were at least 2 ns long.

The side-chain mobility of the polymer was quantified through the Root Mean Square Fluctuation (RMSF) of all but H atoms:
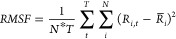
2with *N* the number of atoms (except H), T the total number of configuration in the molecular dynamics trajectory, *R*_i,t_ the Cartesian coordinate of an atom with index i at time *t* and *R̅*_*i*_ the average coordinate of atom i along the trajectory.

## Supporting Information Available

Additional experimental and computational data (PDF)
