# Supplementary material for: Competing Kinetic Consequences of CO2 on the Oxidative Degradation of Branched Poly(ethylenimine)
Source: J Am Chem Soc. 2024 Aug 30;146(41):28201–13. doi: 10.1021/jacs.4c08126 (PMC11487567; doi:10.1021/jacs.4c08126)
Supplement: Supplementary file 1 — ja4c08126_si_001.pdf [file ja4c08126_si_001.pdf]

Supporting Information

Competing Kinetic Consequences of CO_2 on the Oxidative Degradation of Branched Poly(ethylenimine)

Sichi Li,[†,¶] Yoseph Guta,[‡,¶] Marcos F. Calegari Andrade,[†] Elwin Hunter-Sellars,[†] Amitesh Maiti,[†] Anthony J. Varni,[†] Paco Tang,[‡] Carsten Sievers,[*,‡] Simon H. Pang,[*,†] and Christopher W. Jones[*,‡]

†Materials Science Division, Lawrence Livermore National Laboratory, Livermore, CA 94550, United States

‡School of Chemical & Biomolecular Engineering, Georgia Institute of Technology, Atlanta, GA 30332, United States

¶These authors contributed equally to this work.

E-mail: carsten.sievers@chbe.gatech.edu; pang6@llnl.gov; cjones@chbe.gatech.edu

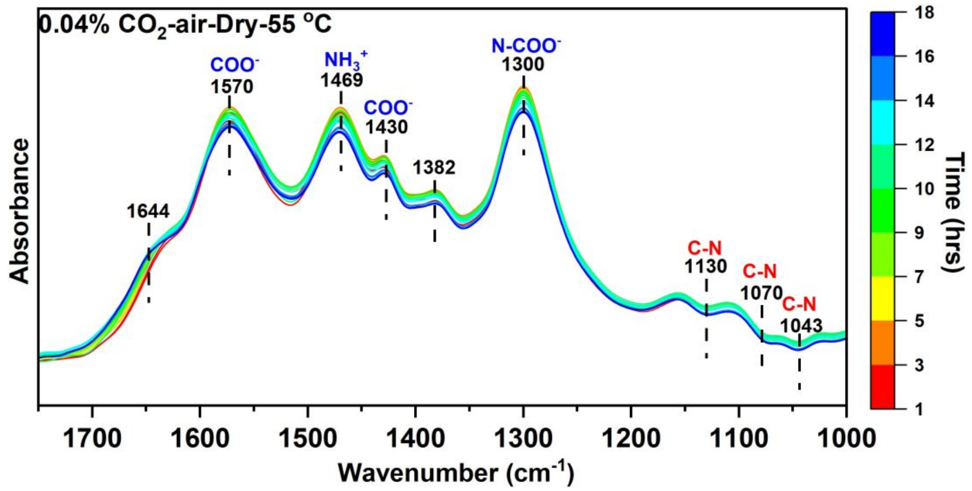

Figure S1. *In situ* ATR-IR spectra (1750–1000 cm^{-1}) of PEI/Al_2O_3 sorbent deactivation under 0.04% CO_2-air at 55 °C for 18 hours

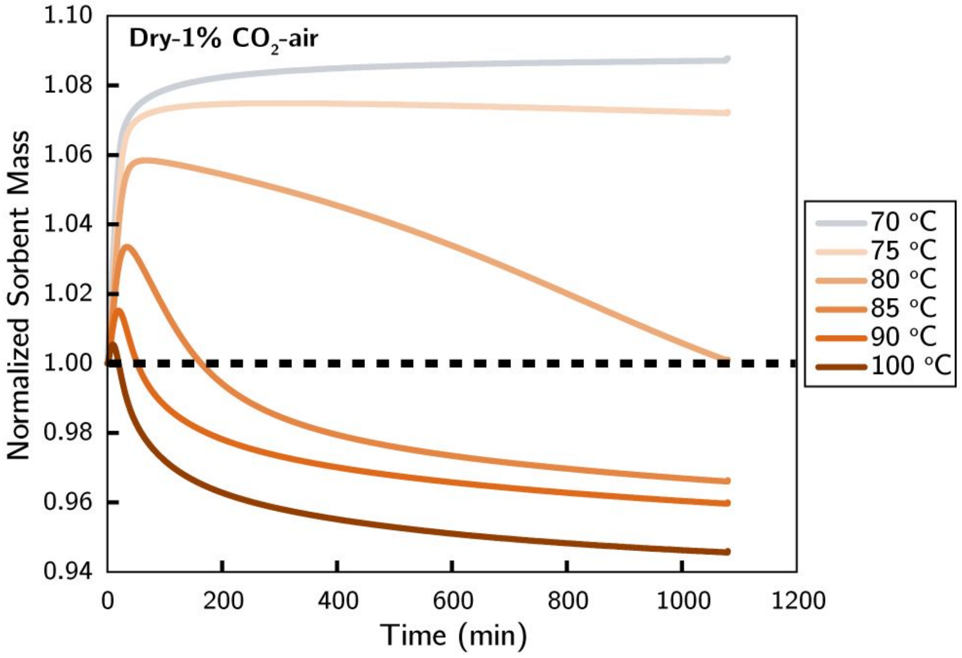

Figure S2. Sorbent mass change, normalized to the initial mass, with time under 1% CO_2-air from 70–100 °C

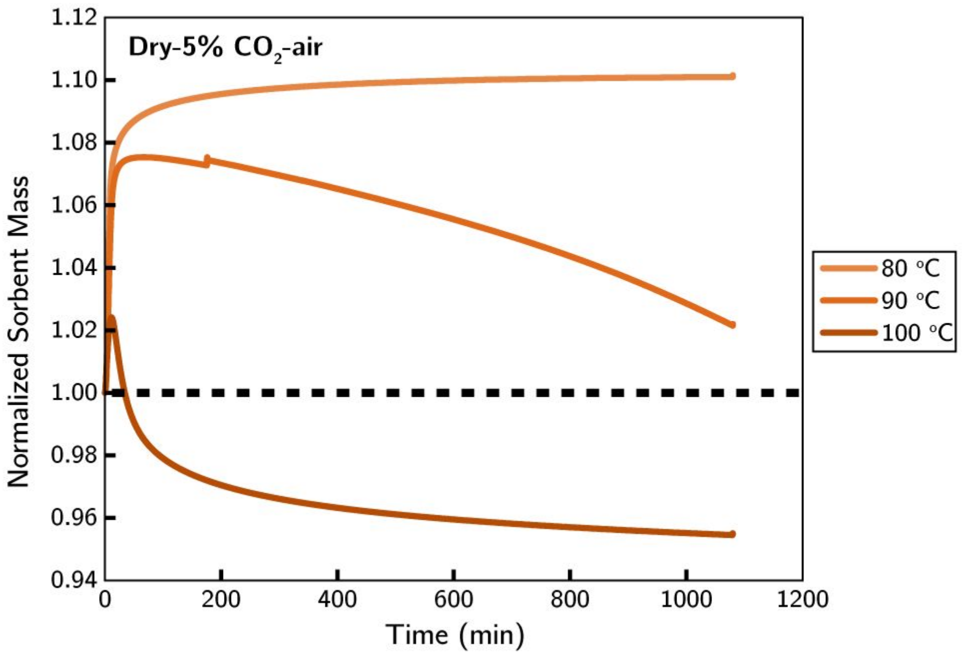

Figure S3. Sorbent mass change, normalized to the initial mass, with time under 5% CO_2-air from 80–100 °C

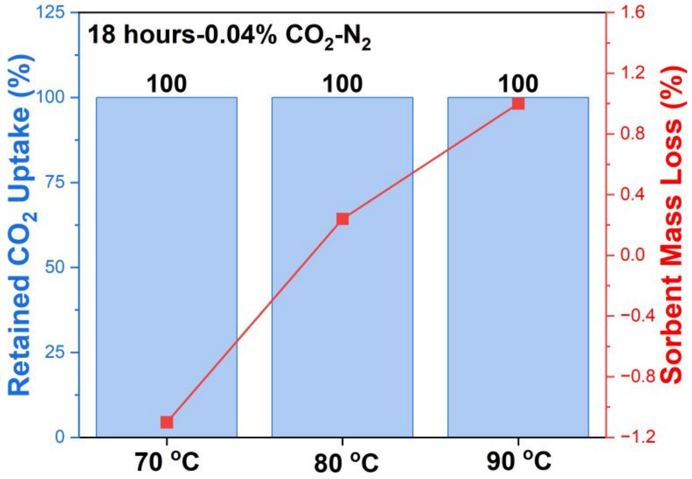

Figure S4. Retained CO_2 uptake, measured at 30 °C, and sorbent mass loss after exposure to 0.04% CO_2-N_2 for 18 hours at 70, 80, and 90 °C

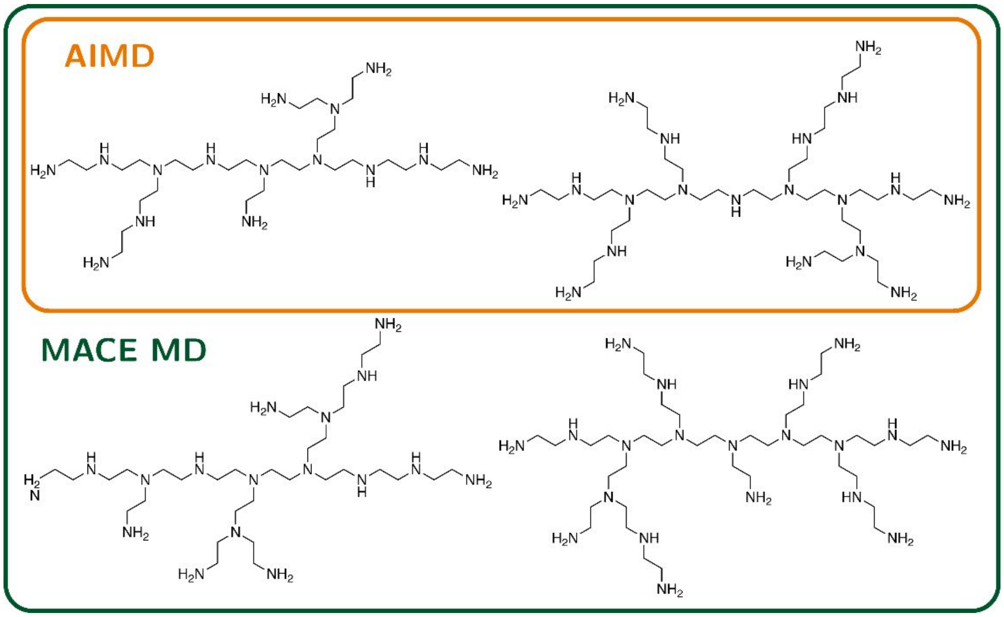

Scheme S1. Model bPEI molecules with structural variations used to construct cubic supercells for AIMD and MACE MD simulations.

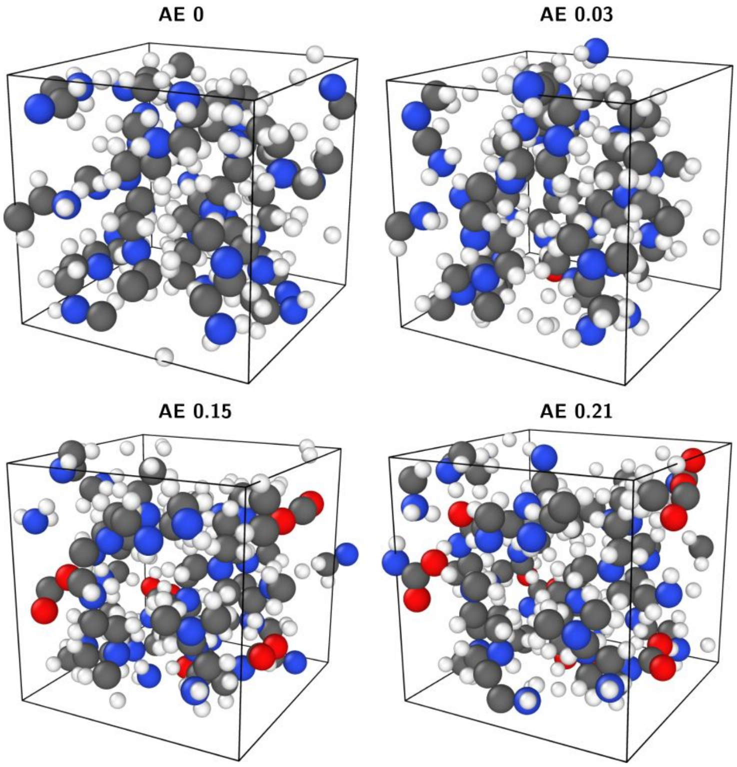

Figure S5. AIMD-equilibrated bPEI simulation supercells with varying numbers of chemisorbed CO_2 molecules corresponding to different amine efficiencies. Color code for atoms: Gray–C, white–H, red–O, blue–N.

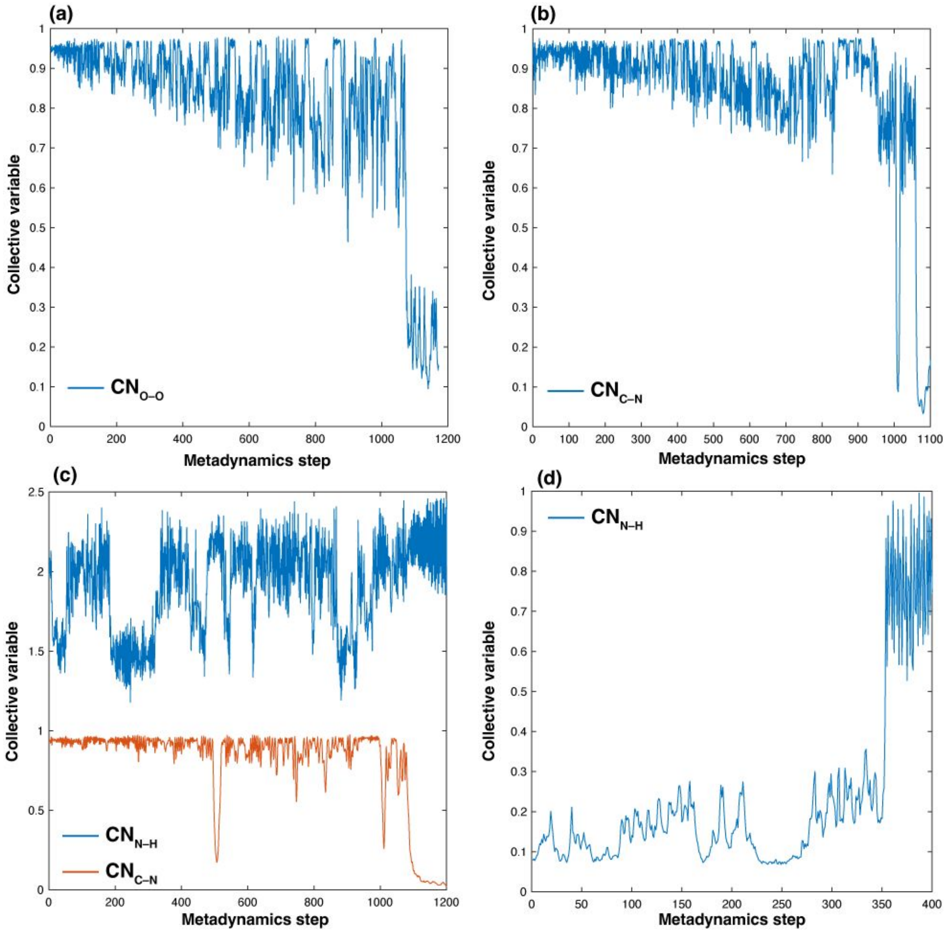

Figure S6. Typical time-evolution profiles of collective variables from metadynamics simulations for: (a) ROOH decomposition, (b) C–N cleavage reaction on CO_2-free bPEI, (c) C–N cleavage on bPEI in the presence of CO_2, and (d) radical propagation through hydrogen abstraction by an aminyl radical.

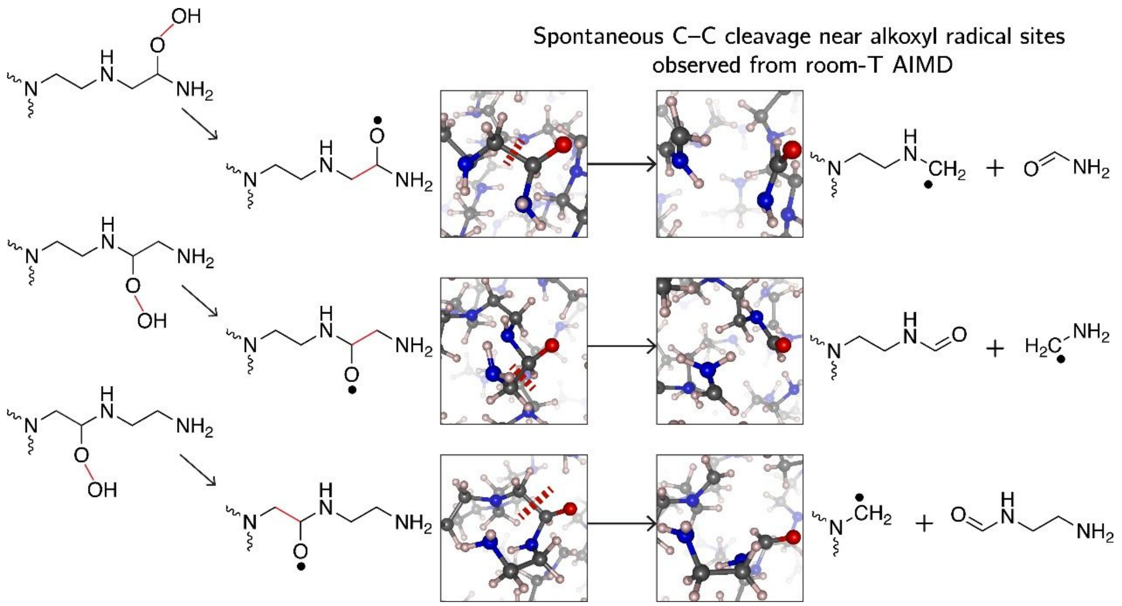

Figure S7. Illustration of spontaneous C–C cleavage pathways following ROOH decomposition. Color code for atoms: Gray–C, pink–H, red–O, blue–N.

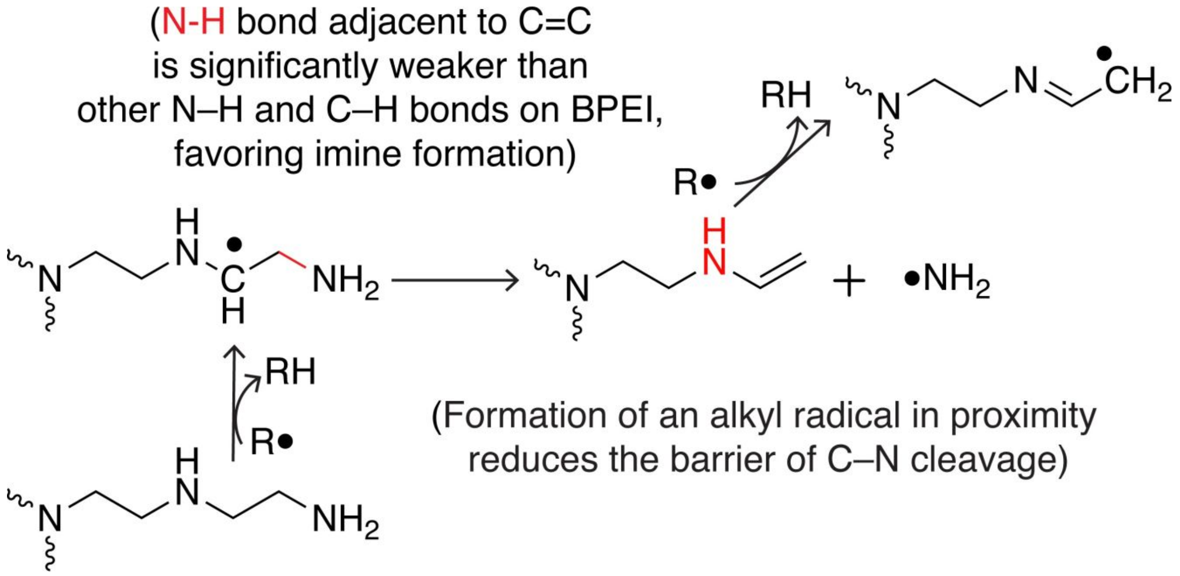

Figure S8. Demonstration of a plausible reaction pathway leading to the formation of an imine product.

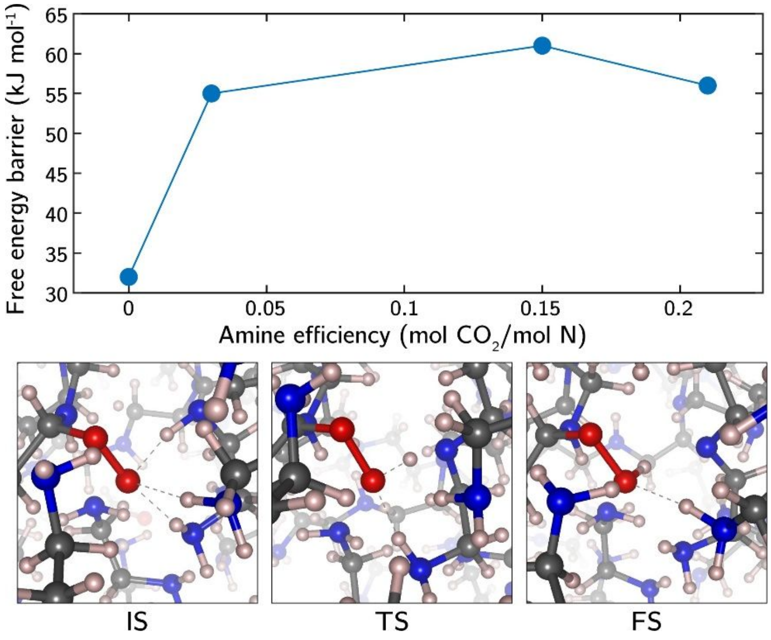

Figure S9. Free energy barriers radical propagation via H abstraction by alkyl peroxyl radicals (ROO•) with varying CO_2 loading (amine efficiency) on bPEI determined from metadynamics simulations at 70 °C. Structures associated with the initial, near-transition, and final states are shown for bPEI with amine efficiency of 0.03 as an illustrative example. Color code for atoms: Gray–C, pink–H, red–O, blue–N.

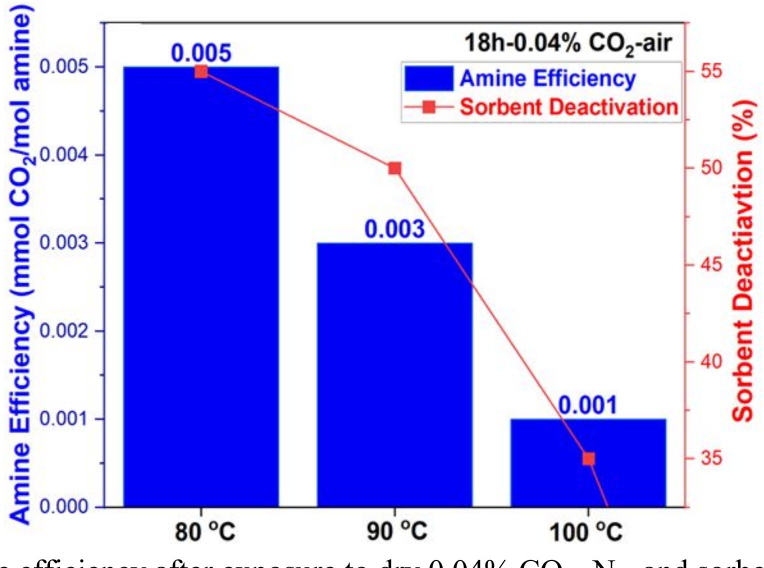

Figure S10. Amine efficiency after exposure to dry 0.04% CO_2 -N_2, and sorbent deactivation (loss in CO_2 adsorption capacity) under dry 0.04% CO_2-air for 18 hours at 80, 90, and 100 °C.

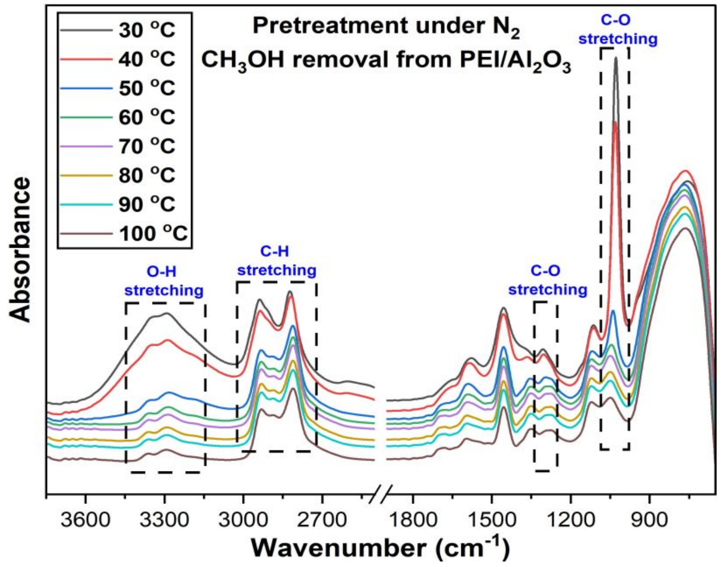

Figure S11. *In situ* ATR-IR spectra (3750–750 cm^{-1}) of PEI/Al_2O_3 sorbent while being heated from 30–100 °C under N_2

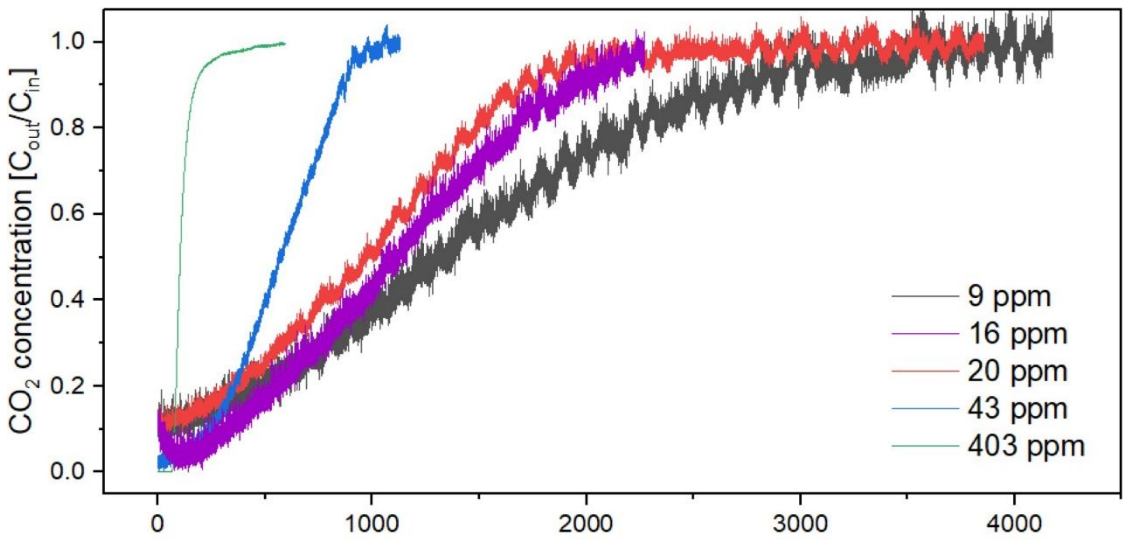

Figure S12. CO_2 adsorption breakthrough curves of PEI/γ-Al_2O_3 at CO_2 concentrations of 9–403 ppm. T = 30 °C.

Table S1. Summary of adsorption and relaxation properties of Al_2O_3-PEI composites.

Exposure conditions [ppm]	Uptake [mmol/g]	Amine Efficiency [mol_{CO2}/mol_N]	T_1 [ms]	T_2 [ms]
0	0	0	38.8 ± 0.7	1.545 ± 0.011
9	0.65	0.075	39.4 ± 0.9	0.846 ± 0.011
16	0.74	0.085	51.3 ± 3.7	0.071 ± 0.001
20	0.80	0.092	60.0 ± 7.1	0.079 ± 0.004
43	1.03	0.118	65.5 ± 4.2	0.067 ± 0.003
414	1.43	0.164	66.1 ± 3.6	0.075 ± 0.003

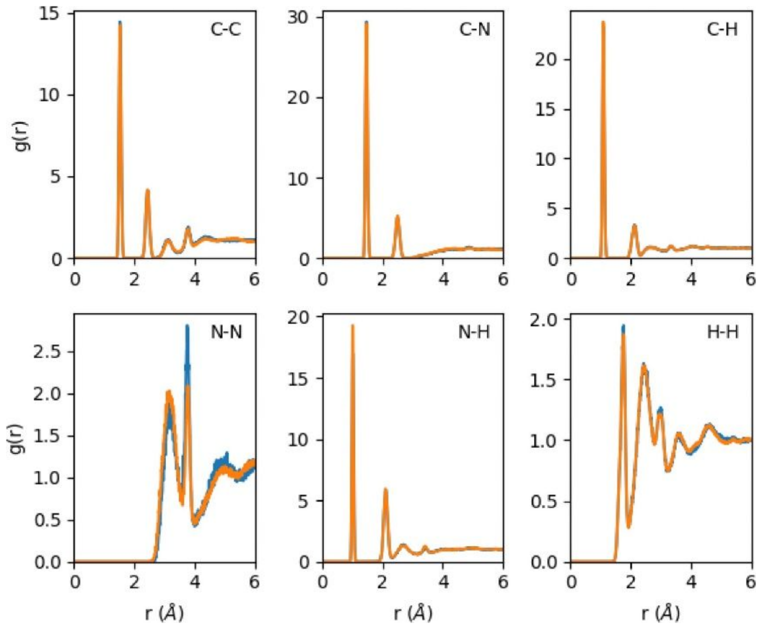

Figure S13. Pair correlation functions derived from trajectories of AIMD compared to MACE-based MD simulations for a CO_2-free 2-bPEI simulation cell.

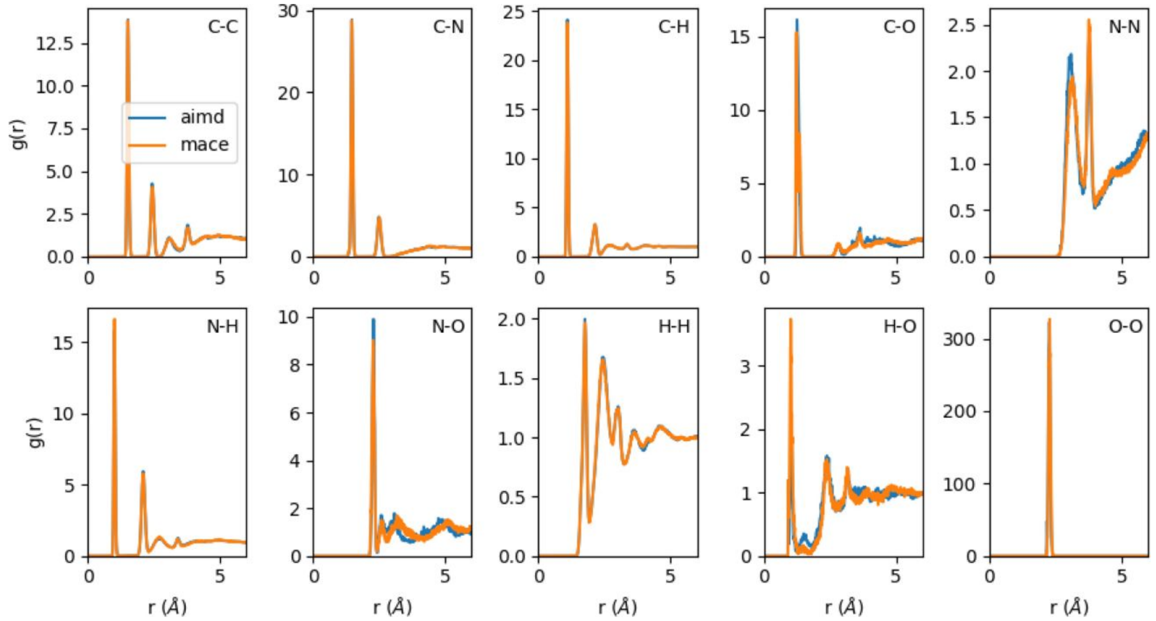

Figure S14. Pair correlation functions derived from trajectories of AIMD compared to MACE-based MD simulations for a 2-bPEI simulation cell containing one chemisorbed CO_2 molecules.

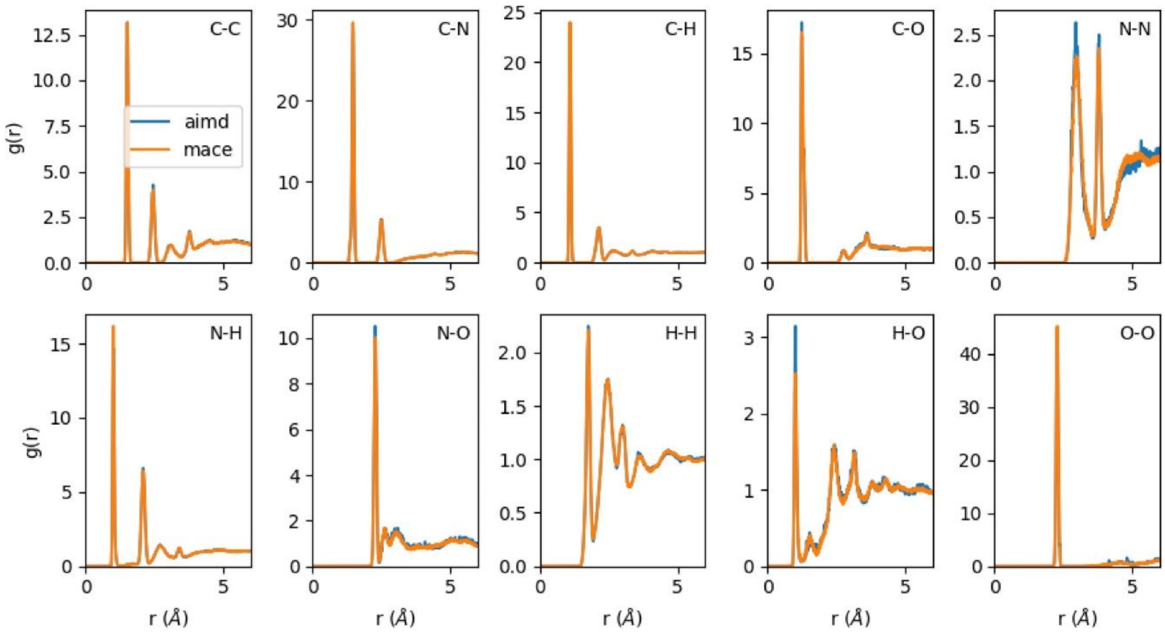

Figure S15. Pair correlation functions derived from trajectories of AIMD compared to MACE-based MD simulations for a 2-bPEI simulation cell containing five chemisorbed CO_2 molecules.
